# The role of environmentally mediated drug resistance in facilitating the spatial distribution of residual disease

**DOI:** 10.1038/s42003-025-08585-9

**Published:** 2025-08-09

**Authors:** Amy Milne, Andriy Marusyk, Philip K. Maini, Alexander R. A. Anderson, Noemi Picco

**Affiliations:** 1 Department of Mathematics, Swansea University, Swansea, UK; 2 Department of Tumor Microenvironment and Metastasis, H. Lee Moffitt Cancer Center, Tampa, FL USA; 3 Wolfson Centre for Mathematical Biology, University of Oxford, Oxford, UK; 4 Department of Integrated Mathematical Oncology, H. Lee Moffitt Cancer Center, Tampa, FL USA

**Keywords:** Cancer microenvironment, Cancer models

## Abstract

The development of de novo resistance is a major disadvantage in molecularly targeted therapies. While much focus is on cell-intrinsic mechanisms, the microenvironment is also known to play a crucial role. This study examines interactions between cancer cells and cancer associated fibroblasts (CAFs) to understand the local crosstalk facilitating residual disease. Using a hybrid-discrete-continuum model, we explore how treatment-induced stress responses can elicit CAF activation and how breaks in treatment allow microenvironment normalisation. We investigate how fluctuating environmental conditions shape the local crosstalk and ultimately drive residual disease. Our experimentally calibrated model identifies environmental and treatment conditions that allow tumour eradication and those that enable survival. We find two distinct mechanisms that underpin residual disease: vasculature-limited drug delivery and CAF-mediated rescue. This work provides a better understanding of the mechanisms that drive the creation of localised residual disease, crucial to informing the development of more effective treatment protocols.

## Abstract (web summary)

Mechanistic modelling of tumour-stroma crosstalk sheds light on how targeted drug dynamics and microenvironmental heterogeneity shape residual disease through distinct mechanisms in the tumour microenvironment.

## Introduction

Among the arsenal of treatments for cancer is molecularly targeted therapy. These drugs act at the molecular level to inhibit pathways that facilitate tumour growth and progression. Examples of molecularly targeted therapies include inhibitors of mutant EGFR and ALK in lung cancers; inhibitors of BRAF in melanomas; or inhibitors of mutant EGFR and HER2 in breast cancer^1–3^. Because of their reduced toxicity due to the selective targeting of a specific mutation, inhibitor targeted therapies are preferable to cytotoxic chemotherapeutic agents which are less tumour specific^4–6^.

Although molecularly targeted therapies offer good initial responses, resistance develops over time and, eventually, treatment fails^6,7^. Mechanisms that drive resistance to targeted inhibitors are still under investigation^5,6^. While the phenomenon of competitive release, where intrinsically resistant cells survive therapy and continue to proliferate due to being freed from the competition of sensitive cells, can explain why treatment fails, it is only one facet of the complexity of drug resistance^8,9^. Interactions between the tumour and the tumour microenvironment (TME) are implicated in the development of a type of resistance dependant on environmental conditions that we call environmentally mediated drug resistance (EMDR)^8,10^. In fluctuating environmental conditions this resistance is transient. Residual disease that occurs from EMDR creates conditions where, in response to the selective pressure of therapy, permanent cell-intrinsic resistance can develop.

The TME is comprised of many types of cells (including fibroblasts, endothelial cells, immune cells), the extracellular matrix (ECM), and signalling molecules^11,12^. The TME plays a pivotal role in the growth of tumours^13–15^, where tumour-TME interactions can both promote^16–18^, or inhibit^19,20^, tumour growth.

Cancer associated fibroblasts (CAFs) are the main producer of ECM and signalling molecules within the TME and can experience morphological and functional changes in the presence of cancer^21,22^. Recent reviews highlight heterogeneity amongst CAF populations in both phenotype and function, the extent of which is an active field of research^21–26^. CAFs also display phenotypic plasticity, where they can switch between phenotypes under specific conditions^27^. These phenotypic changes have been shown to be reversible upon removal of the extracellular cues that drive the changes^28–30^.

Of interest to our investigation of EMDR is the role of CAFs in remodelling the TME and secreting factors essential for the reactivation of proliferation pathways targeted by the inhibitor drug^31,32^. Mechanisms by which CAFs reactivate cancer cell proliferation pathways are varied and include both contact-mediated and non-contact-mediated cancer-stroma interactions^31,33^.

The emergence of reversible CAF-driven EMDR in response to molecularly targeted therapy has been observed experimentally^31^. Introducing breaks in drug delivery, through intermittent treatment regimes, creates fluctuating environmental conditions that dynamically alter extracellular cues in the TME that can delay the onset of resistance^34^. In this paper we will explore the hypothesis that fluctuating environmental conditions can result in transient populations of CAFs, activated through cell-to-cell interactions, which play a role in the response to therapy. Understanding the dynamics underpinning residual disease can crucially allow us to harness the transient nature of TME reactivity to design successful intermittent treatment regimes.

Mathematical modelling has been used extensively to explain the complex dynamics of the TME^35–38^. Some examples include the investigation of the interactions between non-small cell lung cancer (NSCLC) and CAFs under targeted therapy with alectinib using evolutionary game theory techniques^39^ and a multiscale model that characterises the role of CAFs in metastasis^40^. A minimal ODE model integrated with in-vitro and in-vivo experimental data is introduced to quantify the contribution of CAFs to resistance^41^. The significant heterogeneity of TME-specific properties observed in-vivo indicates the role of specific environmental factors in drug resistance. The variation can be explained by spatial constraints within the tissue, stromal support from the TME or some combination of the two. This minimal model does not capture spatial attributes on a single cell scale that drive response to treatment. Since much of the heterogeneity in this response can be attributed to specific TME conditions, we explore the hypothesis that the interactions between cancer and stroma cells in response to environmental and intercellular signals are key drivers of EMDR. Cancer-stroma interactions can be both antagonistic (such as competition for space and resources) and mutualistic (activation of stroma by tumour-produced signals and the EMDR) under therapy. This interplay can lead to non-trivial complex local dynamics and understanding these is important for the design of effective treatment regimes.

Individual agent-based models are extensively used to model biological systems on the cellular level, with a variety of approaches and techniques^36,42–49^. We propose a hybrid discrete-continuum model to investigate how interactions at the cellular level affect tumour growth when undergoing intermittent treatment with targeted therapy. Cells in the model are discrete entities with individual attributes. They follow a set of probabilistic rules that determine their behaviour. This results in complex emerging dynamics at the cell population (tissue) level.

We investigate how the dual nature of local interactions between cancer and stroma cells result in complex emerging dynamics when undergoing molecularly targeted therapy. We explore the potential of introducing treatment holidays to reduce the incidence of EMDR, harnessing the transient and reversible nature of drug-induced changes in the TME. This scenario poses the problem of modelling diffusion and growth dynamics across different temporal and spatial scales: the timescale of cellular processes, and the timescale of intermittent treatment scheduling; the spatial scale of drug diffusion, and the spatial scale of short-range cell-to-cell crosstalk and interactions. The model we present allows us to capture and analyse how all these scales come together to modulate EMDR. We show that local drug diffusion dynamics play a crucial role in the crosstalk between cancer cells and CAFs, and determines the overall response to treatment. We further investigate the resulting residual disease and characterise its spatial features at the tissue and cell scales, which discriminate treatment outcomes. Ultimately, understanding these dynamics will enable new effective ways to control the emergence of resistance brought about by tumour-TME interactions.

## Results

We model the temporal and spatial dynamics of a solid tumour growing in a pre-existing homeostatic non-cancerous tissue, and responding to therapy with an inhibitor drug delivered via the tissue’s vascular system. With our model we set out to explore the implications of the transient and reversible nature of stroma reactivity in the context of intermittent treatment with the inhibitor drug. We first consider a range of intermittent treatment schedules and determine a regime that displays long-term control of tumour burden. We then characterise the residual disease in relation to the temporal evolution of TME and cancer-stroma crosstalk through the course of treatment. Specifically, we focus on co-localisation and density of activated stroma and cancer cells over time. Finally, exploring the diffusion dynamics of the inhibitor drug delivered via the bloodstream and of the molecular signalling in the TME, we establish a link between treatment outcome and local vessel density. Ultimately, our spatial and temporal analysis sheds light on the complex dynamics behind the development of transient resistance observed in tumours undergoing targeted therapy with inhibitor drugs.

### Hybrid discrete-continuum model

**Fig. 1:**
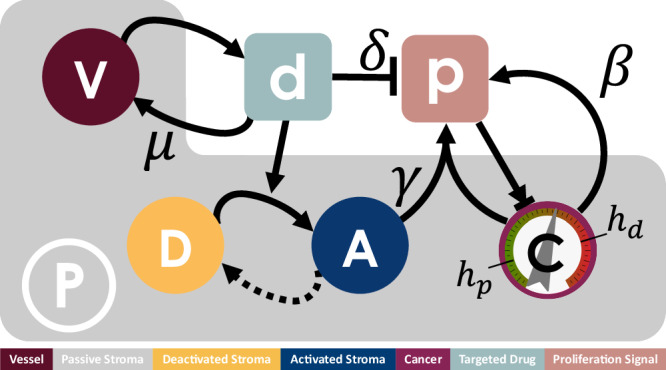
Key interactions between cancer cells and the TME proposed in the model. Cancer cell, , behaviour is dependent on the local concentration of the proliferation signal, , with thresholds for death, *h*_*d*_, and proliferation, *h*_*p*_. Cancer cells provide autocrine promotion of local proliferation signal at rate *β*. TME comprises of both passive, , and reactive stroma. Reactive stroma can be in either an activated, , or deactivated, , state. A targeted inhibitor drug, , depletes proliferation signal at rate *δ* and is removed from the system through vessel sites  at rate *μ*. Local concentration of targeted drug above threshold *h*_*r*_ triggers activation of reactive stroma cells adjacent to a cancer cell, in turn providing paracrine promotion of the proliferation signal at rate *γ*. Activated reactive stroma reverts to a deactivated state if the drug concentration falls below *h*_*r*_.

We adopt a hybrid discrete-continuum model to describe the dynamics of cells acting as individual agents, coupled with the reaction-diffusion dynamics of drug and concentrations of signalling molecules that mediate EMDR (Fig. 1). The modelling framework was first introduced to model nematode movement and chemotaxis^50^.

We introduce the proliferation signal, *p*(**x**, *t*), which represents the local net accumulation of pro-growth versus pro-apoptotic signalling within the tissue^10^. The local signal intensity, sensed by a cancer cell, will determine its viability and proliferative status, based on two thresholds (proliferation above *h*_*p*_, death below *h*_*d*_, and growth-inhibited for intermediate values of *p*(**x**, *t*)).

Modulation of the proliferation signal is provided by the cancer cells, the inhibitor drug, and a subset of non-cancer cells in the TME that are able to engage in crosstalk with the cancer cells when challenged with drug treatment. In our model we refer to all non-cancer cells in the TME as stroma. We designate as reactive the subset of the stroma population that, through cell-to-cell contact, is able to interact with the cancer cells. The experimental counterpart of these cells are CAFs that display some level of differentiation when drug-naive, and a rescue capability to cancer cells in close proximity upon delivery of the drug^31^. The remaining stroma is considered passive.

We introduce a generic inhibitor drug, *d*(**x**, *t*), that is delivered through the blood circulatory system, diffuses through the tissue, and targets a key driver mutation in the cancer cells. The inhibitor drug inhibits pro-growth signalling and enhances pro-apoptotic signalling, ultimately reducing the viability of cancer cells. We assume that reactive stroma proximal to cancer is activated by the local drug concentration, namely when *d*(**x**, *t*) is above threshold *h*_*r*_. Although targeted therapies do not directly affect CAFs (being mutation specific), CAF activation reflects therapy-triggered wound/stress response from tumour cells. In the first instance, we adopt the simplest description of tumour-stroma crosstalk requiring contact-mediated activation (although we explore the effect of non-contact-mediated crosstalk in Supplementary Information S.1). Given that diffusion of signalling molecules is physically limited by ECM and uptake by local cancer cells, it is reasonable to assume highest levels of crosstalk at the tumour-stroma interface. Once activated, reactive stroma provides paracrine production of the proliferation signal that can rescue cancer cells by returning them to a viable, proliferative state. While there is no definite evidence of tumour microenvironment renormalisation after the cessation of treatment, clinical studies show a renewed response upon re-administration of TKI therapies^51–53^. Furthermore, in-vivo data points at the normalisation of the TME following the cessation of inhibitor drug delivery as a possible explanation for re-sensitisation, which, in turn, allows further response upon the reintroduction of treatment^54^. Therefore, activation of reactive stroma in our model is transient and contingent on therapy-triggered recruitment cues from cancer cells.

### Tuning intermittent treatment to control tumour burden and reduce total treatment days

**Fig. 2:**
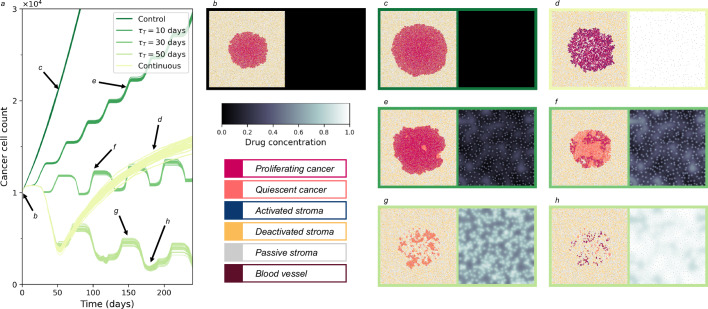
Exploration of treatment scheduling. **a** Tumour burden for *t* ∈ [0, 240] days under no treatment, continuous treatment, and five intermitted treatment schedules (*τ*_*T*_ = {10, 30, 50} days and *τ*_*H*_ = 20 days). Individual realisations are shown, with 30 stochastic simulations conducted for each treatment regime. Reduction in tumour burden is observed as the length of the treatment period *τ*_*T*_ of intermittent treatment increases. Continuous treatment initially displays a very good response to treatment, followed by EMDR-driven relapse. **b**–**h** show spatial distribution and drug concentration at representative time points for a single simulation of the regime of interest. **b** Day 0, the initial condition for all simulations. **c** Day 51 of no treatment. **d** Day 181 of continuous treatment regime. **e** Day 150 of intermittent treatment (*τ*_*T*_ = 10 days, *τ*_*H*_ = 20 days) regime. **f** Day 100 of intermittent treatment (*τ*_*T*_ = 30 days, *τ*_*H*_ = 20 days) regime. **g** Day 150 of intermittent treatment (*τ*_*T*_ = 50 days, *τ*_*H*_ = 20 days) regime. **h** Day 181 of intermittent treatment (*τ*_*T*_ = 50 days, *τ*_*H*_ = 20 days) regime. Animations of the proliferation signal, spatial distribution and drug concentration for each treatment regime are available in Supplementary Information S.5.

We explore alternative treatment scenarios of intermittent treatment (Fig. 2). After detection (*t* = 0) the tumour will continue to grow unless treatment to reduce the tumour is initiated. The tumour burden increases rapidly as cancer invades the homeostatic tissue (Fig. 2c). Alternatively, if the tumour is treated with the inhibitor drug continuously from detection, we observe at first a reduction in tumour burden as the proliferation signal is brought below the proliferative threshold and the bulk of cancer cells become quiescent and die. However, as treatment continues, there is progressive activation of reactive stroma, eventually resulting in overall growth of the tumour (Fig. 2d). Through paracrine promotion of the proliferation signal, reactive stroma is able to rescue a portion of the cancer cells, returning them to a proliferative state. However, the cumulative effect of stroma activation, under continuous treatment conditions, operates on a longer timescale compared to that of proliferation signal depletion. This results in a delay before regrowth is observed, following the initial response. The resulting tissue is composed of a mass of surviving, proliferative cancer cells infiltrated by activated reactive stroma.

The transient and reversible nature of stroma activation, triggered by drug treatment, can be exploited by modulating drug delivery through intermittent treatment. Namely, introducing treatment holidays, where delivery of the inhibitor drug is paused, allows us to control the promoting and rescuing action of reactive stroma. However, reducing overall stroma activation with pauses in drug delivery comes at the expense of tumour burden control. During treatment holidays no additional drug enters the domain while the drug already in the domain diffuses out through the vessels. Eventually, reduced drug concentration leads to deactivation of reactive stroma cells, and hence removal of the local paracrine assistance provided to rescue cancer cells. As a result, those cancer cells that are relying on this paracrine assistance can become quiescent and die. However, cancer cells that survive while local drug concentration decreases sufficiently can re-enter a proliferative state. This can lead to a surge in proliferation over a treatment holiday, albeit hindered by spatial competition from stroma cells that had infiltrated the space freed up by bulk cancer death over the previous treatment delivery. Crucially, the overall outcome of an intermittent regime depends on the prevalence and time scales of all of the processes described above. To investigate this further, we consider intermittent treatment schedules with regular alternating periods of drug delivery and drug holiday. We define *τ*_*T*_ and *τ*_*H*_ to be the length of the drug delivery and drug holiday periods, respectively.

An example of the resulting tumour burden for varying lengths of drug delivery period *τ*_*T*_, with *τ*_*H*_ = 20 days, is shown in Fig. 2. We observe that with short treatment periods (*τ*_*T*_ = 10 days) the drug concentration in the domain is not sufficient to cause significant death in the cancer population (Fig. 2e). For *τ*_*T*_ = 30 days tumour growth is reduced, however the drug concentration in the domain is not sufficient to control the tumour burden (Fig. 2f). Note that, after approximately 150 days, the outcome is comparable to that of the continuous treatment regime. For treatment period lengths greater than 30 days, we observe significant reduction in tumour burden within the first two treatment periods (Supplementary Fig. S2c) and long-term control of tumour burden at low levels (Fig. 2g, h).

**Fig. 3:**
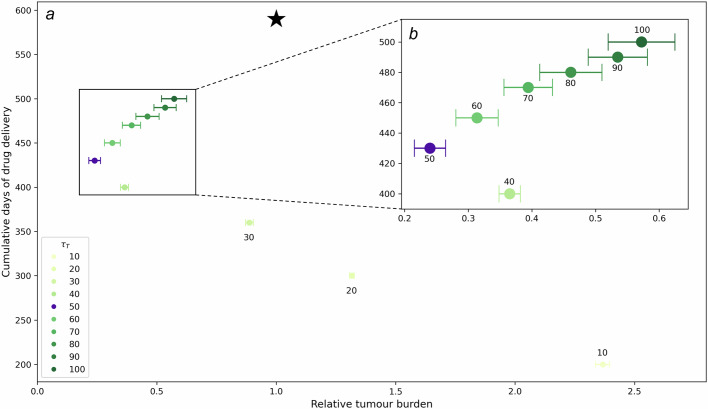
Quantification of treatment regime outcomes. **a** Cumulative days of drug delivery (measured as the sum of drug delivery days over 590 days of therapy) against relative tumour burden (measured as the sum of total cancer cell count over the *t* ∈ [0, 590] day window normalised to the continuous treatment case) for ${\tau }_{T}=\left\{10,20,30,40,50,60,70,80,90,100\right\}$ days and *τ*_*H*_ = 20 days. For each schedule, averages over 30 simulations and 95% confidence intervals are shown. The star indicates reference measures for continuous treatment. As *τ*_*T*_ is increased, relative tumour burden initially decreases and cumulative days of drug delivery increases, but from *τ*_*T*_ = 50 days, the relative tumour burden increases. **b** The inset zooms in on measures for schedules with *τ*_*T*_ ≥ 40 days. The treatment regime *τ*_*T*_ = 50 days, *τ*_*H*_ = 20 days is chosen for further analysis.

To compare the outcomes for different choices of *τ*_*T*_ we extend our investigation of these regimes to a longer window of time. We consider the tumour burden and the cumulative days of drug delivery, relative to the continuous treatment case, over 590 days of therapy. These measures allow us to quantitatively consider the trade-off between reduction of tumour burden and duration of pharmaceutical intervention. Under the parameter regime adopted here, intermittent treatment with *τ*_*T *_≥ 30 days results in lower tumour burden compared to continuous treatment (Fig. 3a). Exploring the 40 ≤ *τ*_*T *_≤ 100 days range further, and considering outcomes over multiple replicates, we observe a non-linear correspondence between *τ*_*T*_ and relative tumour burden (Fig. 3b).

Despite the reduced toxicity of most inhibitor drugs, the cumulative drug delivery time would be a crucial factor in the context of clinical decision-making over treatment scheduling for a patient. We must therefore consider the trade-off between minimizing relative tumour burden and limiting cumulative treatment days. These considerations suggest treatment regimes with 40 ≤ *τ*_*T*_ ≤ 60 provide improved tumour burden while also reducing total number of drug delivery days. A more extensive investigation of treatment regimes (*τ*_*H*_, *τ*_*T*_) can be found in Supplementary Information S.2.

It is important to note that these quantitative results rely on the specific parameter regime adopted (see Table 1 and Supplementary Information S.3 for details of experimentally-informed parameter calibration). In a clinically relevant scenario, some of the model parameters would have to be calibrated against patient-specific measurements.

**Table 1:** Model parameters

Param.	Description	Value	Reference
*h*_*d*_	proliferation signal threshold for death	0.2	Model specific
*h*_*p*_	proliferation signal threshold for proliferation	0.8	Model specific
*p*_0_	proliferation signal at birth	0.256	Experimentally calibrated (see S.1)
*h*_*r*_	inhibitor drug threshold for stroma activation	0.93	Model specific
*D*_*d*_	inhibitor drug diffusion coefficient	10^−5^ cm^2^day^−1^	Extrapolated from^78^, based on radius^79^, for average molecular weight of inhibitor drugs^80^
*β*	proliferation signal autocrine production	1.25 day^−1^	Experimentally calibrated (see S.1)
*γ*	proliferation signal paracrine production	4.242 day^−1^cell^−1^	Experimentally calibrated (see S.1)
*δ*	proliferation signal degradation by inhibitor drug	6.68 day^−1^	Experimentally calibrated (see S.1)
*μ*	rate of inhibitor drug removal at vessel site	500 day^−1^	Model specific
*π*	proportion of reactive to passive stroma	0.5	Model specific
*τ*_*T*_	treatment period length	varies	Model specific
*τ*_*H*_	holiday period length	varies	Model specific
∣Ω∣	domain size	0.3 cm × 0.3 cm	Model specific
*Δx*	lattice spacing	10 *μ*m	Eukaryote cell size^81^
*Δt*	length of timestep	0.044 hours	Model specific
*σ*_mean_	average distance between vessel sites	0.016 cm	^47^
${\sigma }_{\min }$	minimum distance between vessel sites	0.008 cm	^47^
*I*	cell inter mitotic time	*U*[0.9, 1.1] days	^82^
*p*_*T*_	stroma cell turnover probability	0.0042 day^−1^	^47^
*p*_*A*_	stroma cell activation probability	0.042 day^−1^	Model specific
*n*_*CI*_	contact inhibition	2 cells	Model specific

Henceforth, we will adopt the *τ*_*T*_ = 50 days and *τ*_*H*_ = 20 days as the intermittent treatment schedule of choice to investigate how fluctuating environmental conditions modulate transient stroma activation, crosstalk with cancer, and, ultimately, the resulting residual disease.

### Tumour-stroma colocation shapes the emergence of EMDR

**Fig. 4:**
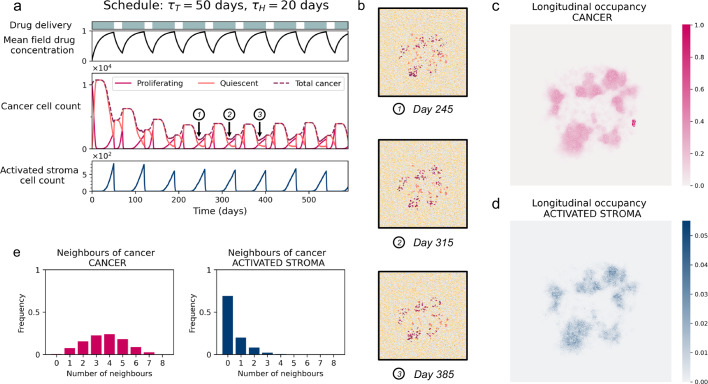
Exploration of spatial attributes of EMDR. **a** Timecourse of a single representative simulation of treatment schedule *τ*_*T*_ = 50 and *τ*_*H*_ = 20 over 590 days. Drug concentration mean field value; cancer cell populations; and activated stroma cell populations. **b** Spatial distribution of cells at 245, 315 and 385 days, corresponding to lowest total cancer cell population in treatment cycles away from the initial transient (corresponding time points are indicated in **a**). **c**, **d** Longitudinal occupancy of cancer and stroma, respectively, in the domain for *t* ∈ [150, 590] days (discarding transient) over 30 simulations. Occupancy is measured as fraction of time a lattice location in the domain is occupied by the cell type of interest. **e** Distributions of average number of cancer cell neighbours of cancer cells and activated stroma in the activation window over the same 30 simulations in (**c**, **d**) (discarding transient) with standard error shown. Here the activation window is the last 60% of the 50 days treatment window. Animations of the spatial distribution, proliferation signal and drug concentrations for treatment regime *τ*_*T*_ = 50 days, *τ*_*H*_ = 20 days are available in Supplementary Information S.5.

With the treatment regime *τ*_*T*_ = 50 and *τ*_*H*_ = 20 we observe control of tumour burden but not eradication (Fig. 2). Figure 4a shows the cell counts of cancer and activated stroma cell types along with the mean field drug concentration. Once the dynamics settle around an approximately cyclic pattern we observe that surviving cancer (i.e. residual disease) is located in similar regions over consecutive treatment cycles. An example of spatial configurations of residual disease at corresponding times of the three consecutive treatment cycles is shown in Fig. 4b. It can be seen that the regions in the domain where the cancer persists are the same for each of the three time points considered.

After discarding the transient window *t* ∈ [0, 150] days, we quantify the longitudinal occupancy of cancer cells over the remainder of the treatment. This measure allows us to determine regions in the domain where surviving cancer is located once the dynamics become approximately cyclic (Fig. 4c). Comparing these regions to those with high longitudinal occupancy of activated stroma (Fig. 4d), we note that they largely overlap. This co-location points to the fact that activated stroma is driving resistance, that is, in these regions we observe tissue-scale EMDR at play. There is, however, a smaller region with remarkably high longitudinal occupancy of cancer which does not correspond to a region of high stroma activation. We will later analyse and compare these distinct regions.

To investigate local cell-to-cell interactions between cancer and stroma, we consider the activation window: the period of the treatment cycle when activated stroma is present. In the treatment regime considered, this corresponds to the end of each drug delivery period, when the inhibitor drug concentration has reached a level sufficient for stroma activation. Moving our analysis from tissue- to cell-scale dynamics, we characterise the makeup of the neighbourhood of cancer cells surviving during the activation window. The distribution of other cancer cells neighbouring a surviving cancer cell is approximately symmetric, with an average of four cancer neighbours in their Moore neighbourhood (Fig. 4e). This represents a shift to the left when compared to the initial distribution of cancer neighbours of cancer cells grown in the homeostatic, drug-free environment (Supplementary Fig. S5). With fewer cancer neighbours providing autocrine signalling, cancer survival depends on paracrine signalling from the TME. Analysing the distribution of activated stroma neighbours around surviving cancer cells during the activation window, we can see that a small number of activated stromal neighbours is sufficient to provide paracrine protection from the effects of the inhibitor drug (Fig. 4e).

### Eradication, survival, and persistence niches

**Fig. 5:**
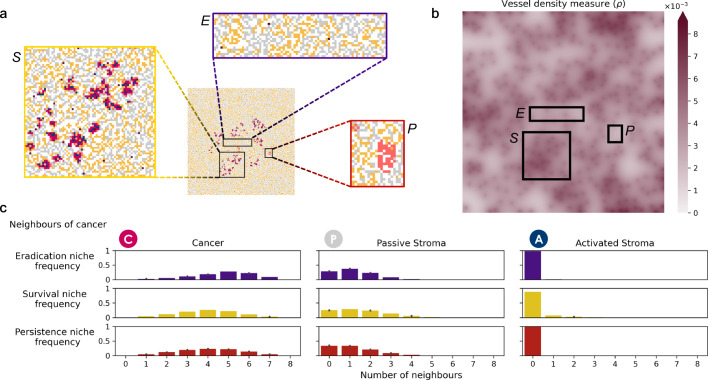
Niche characterisation: cell neighbourhoods and local vessel density. **a** Cell distributions at day 526 from a single representative simulation. Zoomed-in insets are examples of a survival niche (S), an eradication niche (E) and a persistence niche (P). **b** Vessel density measure, *ρ*(**x**), over the domain, for the static vessel distribution ${{{\mathcal{V}}}}$. Here $\hat{\alpha }=0.1$. Boxes tracing the same regions considered in (**a**), show higher *ρ* in the survival niche compared to the eradication niche, and lowest *ρ* in the persistence niche. **c** Distributions of different cell types of neighbours to cancer cells, over the *t* ∈ [0, 590] days window, for the same 30 simulations as Fig. 4. Distributions of average cancer, passive stroma and activated stroma neighbours of cancer cells with standard errors in each niche are shown.

Having observed two different patterns of survival, one driven by co-location of reactive stroma, and the other in the absence of it, we move to fully characterise the TME conditions that allow emergence of resistance. The spatial distribution of residual disease reveals three distinct niches in the domain. Examples of an eradication niche, an EMDR-driven survival niche, and a persistence (non EMDR-driven) niche are shown in Fig. 5a. As the inhibitor drug enters the domain, and concentration builds up, a wave of cancer cell death follows (links to animations are provided in Supplementary Information S.5). Both the survival and eradication niches experience these dynamics of bulk death, whereas the persistence niche does not and cancer cells survive throughout the treatment in a quiescent state. However, in the survival niche small clusters of cancer cells escape the effect of treatment for the duration of the drug delivery window. As the wave of cancer death occurs there is the opportunity for stromal cells to infiltrate this newly accessible space (yet within the constraints of contact inhibition).

To investigate the dual (promoting and competing) nature of cancer-stroma interactions we compare TME conditions in these niches. Firstly, we characterise cancer cell neighbourhoods and find that over the entire treatment window, cancer cells in the survival niche have fewer cancer neighbours and more passive stroma neighbours, when compared to those in the eradication niche (Fig. 5c). These observations point to reduced autocrine assistance and more spatial competition from passive stroma neighbours, respectively. A similar result is observed when considering spatial competition from reactive stroma (irrespective of activation status, Supplementary Fig. S6). Reduced autocrine signalling and increased spatial competition are features that we would intuitively attribute to an eradication niche, rather than a survival niche. However, when analysing the activated stroma neighbours of cancer cells in both niches, we can clearly see that cancer cells in the survival niche experience higher paracrine promotion over the course of treatment. Therefore, we find that it is the paracrine stimulus to proliferation in the survival niche that can shift the modulation of proliferative signal and enable survival and growth, making up for loss of autocrine promotion and enhanced spatial competition. We note that in the persistence niche the distribution of cancer neighbours (hence the autocrine signalling to the average cancer cell) is approximately similar to the one in the survival niche. However, given the complete absence of activated stroma in the neighbourhood of cancer cells, paracrine promotion does not explain survival in the persistence niche. We will next identify other mechanisms at play that can explain survival in the persistence niche.

Since all niches emerge from homogeneous conditions (i.e. the same initial mass of cancer cells immersed in comparably reactive stromal tissue), we look at the inhibitor drug intermittent delivery to identify the source of homogeneity-breaking. Local build up of the drug concentration can induce both cancer cell death, and activation of reactive stroma proximal to cancer. This suggests that the different outcomes depend on the inhibitor drug concentration which, in turn, is determined by vessel distribution and diffusion dynamics. Where the density of vessels is higher, the local concentration of the inhibitor drug will build up quicker compared to where vessel density is lower.

We hypothesise that cancer cells in regions of high vessel density experience more intense effects of the inhibitor drug but are also more likely to be rescued by activated stroma. Figure 5b shows the vessel density measure, *ρ*, for the given vessel distribution, showing that higher density correlates with survival, when compared to the density in the region identified as the eradication niche. This is consistent with our hypothesis that cancer survival depends on vessel density. For low vessel density the local targeted drug concentration will not reach a level sufficient to kill cancer cells within the drug delivery period. As the vessel density increases the local targeted drug concentration will become sufficient to cause bulk death of cancer cells until, for even higher vessel densities, the local targeted drug concentration will reach the threshold *h*_*r*_, giving more time for stroma activation and assistance over each cycle of drug delivery. This protective action will unfold over the long timescale of consecutive treatment cycles. However, we expect this to be a non-linear effect. Excessively high vessel density will facilitate higher drug concentrations during drug delivery and lead to more cancer cell deaths on a much shorter timescale. On the flip side, extremely low vessel density will result in insufficient build up of drug concentration, and reduced cell death. Notably, vessel density in the persistence niche is smaller than that in the eradication niche. Therefore, while stroma activation was identified as a key mechanism behind residual disease in the survival niche (Fig. 5c), low vessel density can explain residual disease in the persistence niche.

### Vessel density driven trade-off in treatment outcome

**Fig. 6:**
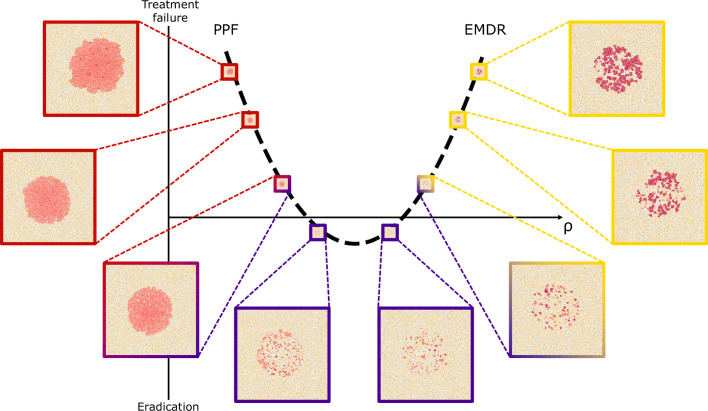
Investigation of vessel density and treatment outcomes. Distribution of cells at day 139 from single representative simulations with increasing vessel density. Vessel sites are determined to reflect a target density across the domain (see Supplementary Information S.6 for details). Increasing mean field *ρ* values are 0.54 × 10^−3^, 1.63 × 10^−3^, 2.18 × 10^−3^, 2.73 × 10^−3^, 3.28 × 10^−3^, 3.82 × 10^−3^, 4.38 × 10^−3^ and 4.94 × 10^−3^. Treatment failure due to poor perfusion of the drug (PPF) is evident for low vessel density, while EMDR drives treatment failure for higher vessel densities.

To further investigate how vessel density affects the two antithetic processes of stroma activation and cancer death during treatment, we consider an experiment where intermittent treatment is applied to domains of varying vessel densities. These domains are obtained by systematically decreasing the spacing of vessels placed on a regular grid; all details of this setup are discussed in Supplementary Information S.6. At corresponding times across simulations we observe different responses to intermittent treatment. At low vessel densities, the targeted drug enters the domain at fewer locations and hence is not able to sufficiently diffuse and build up throughout the domain, resulting in cancer cell survival. We call this type of treatment failure, poor perfusion failure (PPF). Figure 6 shows instances of PPF for low vessel densities (lower mean field *ρ* insets colour-coded red and Supplementary Fig. S7 for additional time resolution).

As vessel density increases, the inhibitor drug enters the domain at more locations and is able to diffuse and build up sufficiently to cause bulk death of cancer cells without causing significant activation of stroma. This results in eradication of cancer cells, indicative of treatment success (centre insets colour-coded purple of Fig. 6 and Supplementary Fig. S7 for additional time resolution).

At greater vessel densities, the inhibitor drug enters the domain at more locations and is able to diffuse and build up quickly in the tissue. Reactive stroma is then much more likely to activate and provide the additional paracrine promotion of the proliferation signal required to rescue cancer cells. This results in EMDR (higher mean field *ρ* insets colour-coded yellow of Fig. 6 and Supplementary Fig. S7 for additional time resolution).

Remarkably, the persistence and survival niches identified in previous simulations with a realistic irregular vessel distribution, display outcomes suggestive of PPF and EMDR, respectively.

### Drug dynamics shape distinct niches

**Fig. 7:**
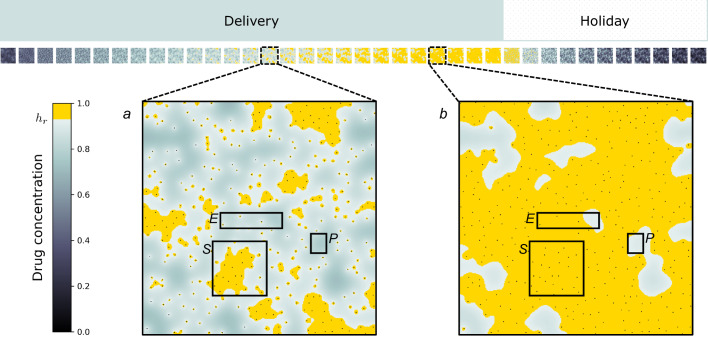
Targeted drug spatio-temporal dynamics over a treatment cycle. Drug concentration, *d*, for the third treatment cycle (drug delivery + holiday period) with yellow highlight for locations where *d* ≥ *h*_*r*_. Representative eradication (E), survival (S) and persistence (P) niches are the same as in Fig. 5. **a** Drug concentration in the middle of the treatment period. The survival niche has a larger fraction of above-threshold locations. The eradication niche has a very low fraction of above-threshold locations and the persistence niche has no locations above the threshold. **b** Drug concentration near the end of the delivery period. Above-threshold locations now cover the entirety of the survival niche, and a large fraction of the eradication niche. There is a very small fraction of locations above the threshold in the persistence niche.

Having observed a transition from PPF to EMDR as the vessel density and/or drug delivery period increases, we further investigate conditions of vessel density and treatment scheduling that can modulate resistance. During one cycle of treatment (one drug delivery period followed by one drug holiday period) the drug concentration builds up in the domain with regions of high local vessel density surpassing the threshold *h*_*r*_ quicker than regions with low local vessel density. Figure 7a shows a snapshot of the drug concentration field just after halfway through the drug delivery period. At this point, a significant fraction of the survival niche experiences drug concentrations sufficient for stroma activation (*d*(**x**, *t*) ≥ *h*_*r*_), while only a very small portion of the eradication niche and none of the persistence niche experience this condition. Later in the drug delivery period everywhere in the survival niche is now above the threshold, while a significant fraction of the eradication niche and a very small portion of the persistence niche are now at drug concentrations sufficiently high for stroma to become activated (Fig. 7b). We argue that low overall drug concentrations result in residual disease as they are not sufficient to cause cancer cell death and long exposure to above-threshold concentrations over each delivery cycle results in increased stroma activation, tipping the balance between the death-inducing and activation-promoting action of the drug, in favour of the latter.

Since a significant fraction of locations in the survival niche experience drug concentrations above *h*_*r*_ for a longer time than the eradication niche, the probability of stroma activation is higher in the survival niche than in the eradication niche. This agrees with the results of longitudinal analysis of activated stroma neighbourhood (Fig. 5c) and activated stroma occupancy (Fig. 4d). On the other hand, the much slower build up of drug concentration in the persistence niche results in local drug concentrations that are not sufficient to kill cancer cells within the drug delivery window. This agrees with results of longitudinal cancer occupancy analysis (Fig. 4c). Insufficient drug concentrations in the persistence niche result in less decay of the proliferation signal and cancer cells do not die within the drug delivery window. Without the added paracrine promotion of the proliferation signal from activated stroma cells, cancer cells in the eradication niche are not able to survive. Conversely, the additional promotion of the proliferation signal provided by the paracrine signalling from the activated stroma in the survival niche enables survival of cancer cells, and ultimately the emergence of EMDR.

### Dormancy and sustained proliferation as distinct mechanisms for survival

**Fig. 8:**
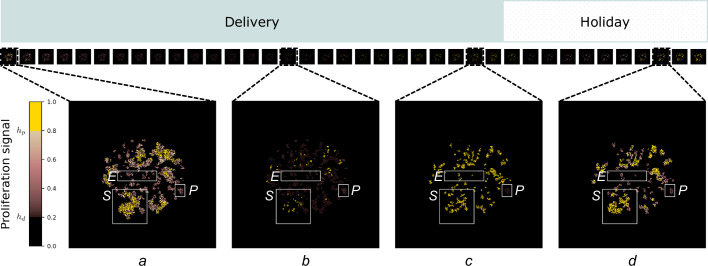
Proliferation signal modulation over a treatment cycle. Proliferation signal, *p*, during the third treatment cycle with yellow highlight for locations where *p* ≥ *h*_*p*_ (proliferation window) and black highlight for locations where *p* < *h*_*d*_ (death window). Representative eradication (E), survival (S) and persistence (P) niches are the same as in Figs. 5 and 7. **a** At the start of the drug delivery period all three niches contain locations where the proliferation signal is above *h*_*d*_, forming a bulk region in the survival and persistence niches, and small sparse clusters in the eradication niche. Regions in the proliferative window are only present in the survival niche. **b** In the middle of the drug delivery period a small number of locations in the survival niche remain in the proliferative window. The proliferation signal in the eradication and persistence niches is significantly depleted, with no locations in the proliferative window. **c** By the end of the drug delivery period in the survival niche the region in the proliferative window has increased through cancer proliferation and further stroma activation. The eradication niche is almost entirely in the death window, although limited regions in the proliferation window indicate late activation of stroma. There are no such locations in the persistence niche, which is largely in the quiescent window. **d** Towards the end of the drug holiday period, in the eradication and persistence niches no locations are in the proliferative window. In the survival niche the region in the proliferative window which appeared during drug delivery expands further, while other locations move from the death to the quiescent window.

Lastly, we consider the cumulative effects of consecutive rounds of drug delivery periods in shaping the proliferation signal, and ultimately the TME landscape which determines cell fate (survival or death) locally, and residual disease at the larger tissue scale. To do so we analyse the third treatment cycle of the intermittent treatment schedule (Fig. 8).

At the beginning of this drug delivery period residual disease is present in each of the three niches. In the eradication niche there are small clusters of locations where the proliferation signal is above threshold for cancer cell death *h*_*d*_. This is quite different to the proliferation signal landscape in the survival and persistence niches where the locations where the proliferation signal is above *h*_*d*_ form a bulk mass.

When treatment commences the drug concentration quickly builds up in the survival niche depleting the proliferation signal. This results both in death at locations away from vessels, and stroma activation followed by a rebound in proliferation signal levels in regions closer to the vessels. This rebound is sufficient to sustain high proliferation signal levels well into the holiday period, despite stroma deactivation. Local paracrine promotion of the proliferation signal is hence the driver for residual disease in the survival niche.

In the eradication niche the drug concentration builds up slowly over the drug delivery window. This translates into a slower depletion of proliferation signals to levels that can trigger cell death, as well as delayed stroma activation. The results of this slower timescale of stroma activation results in sparse cancer cell survival. Residual disease at the beginning of the holiday window is limited and decreases over the following treatment cycles, eventually wiping out the cancer cell population. Lack of paracrine support to proliferation signal is therefore the cause for successful eradication in this region.

In the persistence niche the drug diffusion dynamics are even slower, resulting in drug concentrations that are not sufficient to effectively deplete the proliferation signal. Here, proliferation signal is solely reliant on autocrine signalling produced by clusters of cancer cells. Signal levels remain low but above *h*_*d*_ throughout the treatment cycle, allowing cancer cells to survive the treatment in a quiescent state, and build up some reservoir of proliferation signal over the holiday window. Treatment escape by dormancy is therefore the driver for residual disease in the persistence niche.

## Discussion

The discovery and development of molecularly targeted therapies provide alternative treatment options for cancer patients with the benefit of lower toxicity than cyctoxic therapies. Unfortunately, the success of these therapies has not been as positive as anticipated due to residual disease and the emergence of resistance^13,54–56^. This resistance is not only driven by the evolution of cancer cell intrinsic mechanisms but also due to interactions between the tumour and the Tumour Microenvironment (TME), known as Environmental Mediated Drug Resistance (EMDR). A characteristic of EMDR is reversibility^8^, which can be exploited as a treatment strategy^34,41,51^. However, response to treatment varies between and within individuals, mirroring the heterogeneity of tissue observed in-vivo^41,51^. Therefore, understanding the spatio-temporal dynamics resulting in the emergence of resistance and, specifically, TME conditions that favour residual disease, has the potential to inform future treatment protocols.

Our model is rigorously calibrated and validated against experimental data from mouse xenograft models. It captures the dynamics of EMDR, including the initial response to treatment followed by the resurgence of disease^8,39,54,57^. This resistance is in part driven by paracrine signalling from proximal stroma activated by the tumour in response to drug treatment. When the treatment is withdrawn the stromal response is reversed and in the absence of the inhibitor drug the tumour can resume growth from the few remaining cells in the survival niches. When treatment recommences there is a diminished response, which indicates resistance mechanisms are at play. During intermittent treatment the activated stroma population is transient and depends on drug concentration and cancer proximity. Our in-silico results show that removal of paracrine signalling from the activated stroma during the holiday period can be used to modulate tumour growth, reminiscent of adaptive therapy strategies that exploit intracellular competition to delay the onset of disease progression^41,55,58,59^. Given that cancer cell proliferation and stroma deactivation is governed by dynamics of diffusible signalling molecules and drug delivery and clearance, paracrine signalling does not abruptly vanish at the introduction of a holiday period (Fig. 2a). Therefore, the long-term benefits of the introduction of drug holidays can be appreciated over an intermittent treatment schedule composed of several treatment cycles. We used an intermittent drug delivery regime that can maintain tumour burden in the long-term, as a test-case to investigate the spatial attributes of emergent niches of residual disease. Specifically, we investigated how fluctuating environmental conditions modulate drug perfusion, transient stroma activation, crosstalk with cancer, and, ultimately, the resulting residual disease.

The dual nature of the relationship between the tumour and the TME includes both competition for space (cancer - stroma) and unidirectional cooperation through cues essential for survival (cancer - activated stroma). Additionally, the behaviour of individual cells and cell-cell interactions are dictated by diffusible signalling molecules and the inhibitor drug. The latter can directly result in cell death, as well as stroma activation, thus indirectly offering a route for cancer survival. Non-trivial, complex local dynamics emerge as a result of cellular and molecular processes occurring across different temporal and spatial scales: the timescale of cell birth, death, and state changes; the timescale of intermittent treatment scheduling; the spatial scale of drug diffusion; and the spatial scale of short-range cell-to-cell crosstalk. Our model demonstrates that the interplay between these processes ultimately shapes the heterogeneity of the response to treatment and, ultimately, residual disease.

We have shown how heterogeneity of local tissue conditions can drive different responses to treatment and shape residual disease. Our extensive investigation of longitudinal occupancy and neighbourhood distribution, as well as vessel density analysis, allow for characterisation of niches where cancer cells survive the course of intermittent treatment with targeted inhibitors (Figs. 4 and 5). We have found a non-linear relationship between vessel density and treatment outcome (Fig. 6). Poor treatment outcome can be observed both in regions of low vessel density and regions of high vessel density. Resistance is of a different nature, and can be attributed to poor perfusion of the drug (PPF), and high stroma activation (EMDR), respectively. A heterogeneous TME can result in occurrences of both types of resistance. In our in-silico tissue with an irregular, experimentally calibrated, vessel distribution we have identified persistance niches. Here, drug perfusion is insufficient to cause bulk death, due to low vessel density; residual disease is characterised by clusters of largely dormant cancer cells relying solely on autocrine signalling for survival (Figs.5 and 8). In the same tissue we have also observed survival niches emerge. These are regions characterised by high vessel density. Here, the drug can diffuse and build up sufficiently to kill cancer cells exposed to the highest concentrations. However, exposure to moderate drug concentrations triggers signalling from cancer cells experiencing stress to cue rescue from the stroma. We have found that, ultimately, long-term exposure to drug over repeated treatment cycles results in residual disease characterised by active stroma infiltration, and reliance on paracrine signalling for survival (Figs. 7 and 8). Finally, we have observed eradication niches. These regions are characterised by intermediate vessel density, whereby the timescale of drug diffusion being smaller than that of stroma activation results in a complete eradication of cancer (Figs. 5 and 8). These findings show that awareness of TME architecture in terms of both the vascular and stromal structure is central to our ability to improve targeted therapy outcomes. We have highlighted the range of emerging behaviour from the complex interplay between various spatial and temporal scales. While some of those scales cannot be easily controlled (e.g. vessel distribution) without introducing additional treatment (e.g. therapy targeted at vasculature remodelling), others such as the scheduling of intermittent treatment are natural candidates for clinically actionable future directions of this work. Namely, our experimentally calibrated in-silico model has shown that modulating drug concentration through intermittent treatment can reduce the occurrence of EMDR and successfully maintain control of tumour burden whenever eradication is not possible (Figs. 3 and 5).

Adaptive therapy scheduling could be considered, where real-time patient response can inform drug administration protocols^58^. Often the treatment plan for a patient with molecularly targeted cancers includes more than one therapeutic drug, including cytotoxic agents, immunotherapy drugs, and multiple molecularly targeted therapies that target both cancer and stroma^6,31,60,61^. Combinations of therapies could be incorporated into our model to address recent in-vivo observations of stromal proximity being associated with increased proliferation of tumour cells in chemotherapy treatment holidays^44^. The in-silico spatial dynamics of cancer undergoing multiple treatments could inform us as to why such treatment protocols might work or fail.

While the residual disease in persistent niches is maintained in a quiescent state, survival niches provide safe havens for proliferating cancer cells while undergoing treatment. The survival of these cells, despite hostile conditions, can provide opportunities for permanent acquired resistance to develop. Our model could be extended to include evolutionary dynamics for cancer cells, where their response to selective pressures can lead to the development of acquired resistance.

Similarly, while we consider a particular phenotype of CAF, it is important to note that our model can be generalised or extended to investigate interactions between the tumour and other cells in the TME. The passive stroma compartment consists of all cells in the microenvironment other than those considered in this paper, some of which can engage in crosstalk with cancer cells. For example, immune cells can exhibit the same dual role of promoting cancer survival while also competing for space and resources^43^. The CAFs we consider in the model have a specific location, in relation to the tumour, and function. Given the plasticity of the CAF phenotype^21–23,25^, a less trivial extension of our model could include alternative CAF phenotypes and the transition between them^24^. For example, in pancreatic ductal adenocarcinoma, two CAF phenotypes are identified that occupy distinct locations in relation to the tumour and vary in function^28^. Additionally, in neuroblastoma, cancer-stroma crosstalk leading to EMDR has been shown to rely on non-contact-mediated mechanisms^33,62^. While preliminary exploration of alternative hypotheses of activation and deactivation mechanisms has not shown a qualitative change in the dynamics (Supplementary Information S.1), quantitative effects must be accounted for in tumour-type-specific adaptations of this model. Specifically, we have found non-contact-mediated crosstalk results in primed regions of active stroma which significantly enhance tumour growth during treatment holidays (Supplementary Fig. S1).

A limitation of our model is that we assume a simplified TME. The vasculature is 2D perpendicular to the tissue, and we do not consider the upregulation of extracellular matrix secretion upon stroma activation^63^, both of which affect drug diffusion dynamics. Potential extensions to our model could incorporate more complex vasculature (non-perpendicular or 3D) or stroma-driven drug protection mechanisms. Similarly, extending the model to include angiogenesis would allow for the investigation of these complex dynamics in tumours characterised by a dynamic vasculature, and vascular treatments such as anti-angiogenesis, like bevacizumab or sunitinib^64^.

Our study has primarily been concerned with the spatial attributes that promote the emergence of survival niches when undergoing intermittent treatment with molecularly targeted therapies. We established that the creation of these niches depends on the interplay of local drug concentration and cell-cell crosstalk. Local drug concentration depends on the drug supply (duration of administration) and the physical configuration of drug delivery (vessel distribution). In regions that receive high concentrations of the inhibitor drug, the tumour is under a greater degree of stress. This triggers cues for stromal assistance to aid with survival that are not evident in regions where the tumour is experiencing less stress. Our analysis highlights the importance of considering how the spatial features of tumours and their immediate microenvironment can synergise to drive drug resistance.

## Methods

### Model definition

We consider a rectangular two-dimensional cross-section of tissue that we model with a square lattice, Ω. The domain, Ω, contains discrete agents representing the cells in a cross-section of tissue. In the model we have three sets of agents: blood vessels, stroma and cancer cells.

Let *p*(**x**, *t*) be the proliferation signal and *d*(**x**, *t*) the inhibitor drug concentration, where both *p* and *d* are continuous functions of space, **x**, and time, *t*. Both *p* and *d* are governed by partial differential equations (PDEs), whose formulation depends on the agents in Ω. The concentrations of *p* and *d* are updated by numerically solving the PDEs using a forward difference scheme in time and a second order central difference scheme in space. Cancer and stroma cells then advance through the cell cycle, divide, die and update their state (according to the rules described below) dependent on local concentrations of *p* and *d*. Finally, based on the current configuration of the agents in Ω, the concentrations of *p* and *d* are updated. We impose zero-flux Neumann boundary conditions on the boundary of Ω. To comply with stability requirements of the numerical scheme we solve the equation for *d* on a finer time grid (see Supplementary Information S.7 for details).

Model implementation and simulations are carried out using the Hybrid Automata Library (HAL), a Java Library designed for hybrid modelling of cancer^65^. HAL has been used to simulate models developed to investigate cellular systems in oncological applications^44,66–69^, and other biological contexts^70–72^.

In order to investigate the dynamics of resistance that can be attributed to immediate crosstalk between cancer and stroma cells, we assume that cells do not actively move within the domain but do spread through proliferation. Cancer and stroma cells have an intermitotic time, *I* > 0, and each cell is equipped with an internal clock to track its progress through the cell cycle. Once a cell’s internal clock has reached *I*, cell division occurs only if there is sufficient space (i.e. at least one empty position in the Moore neighbourhood of the dividing cell). Additional conditions for cell division are cell type specific and context dependent (i.e. TME-dependent), as described later. At cell division two daughter cells are produced. One daughter cell occupies the mother’s position and the other daughter cell is placed, at random, in one of the empty locations in the neighbourhood of the mother cell.

All model parameters are described in Table 1. Parameter values were obtained by rigorous calibration based on experimental observations and quantifications, as described in Supplementary Information S.3.

### Blood vessels and drug concentration

Blood vessels are assumed to cut the plane of Ω perpendicularly and to occupy a single lattice position. These are simplifying assumptions, as in reality we would expect a cross-section of tissue to cut through a more intricate vasculature network, with resulting blood vessel positions on Ω comprised of more than one endothelial cell^73^. However, as described later, this simplification still allows us to describe realistic drug diffusion dynamics in the tissue. Our model describes tumour growth prior to angiogenesis, so vascular remodelling and degradation are neglected and blood vessels are static and constant. Vascular cells act as point sources and sinks for the inhibitor drug.

We define ${{{\mathcal{V}}}}$ to be the set of vessel locations, that is ${{{\bf{x}}}}\in {{{\mathcal{V}}}}$ if there is a vessel at position **x**. The circle packing algorithm^47^, is used to determine ${{{\mathcal{V}}}}$. This algorithm uses in-vivo measurements for the average distance between vessels, *σ*_mean_, to calculate the number of vessels in the domain, denoted by *N*_*v*_, where

$${N}_{v}=\frac{| \Omega | }{{{\sigma }_{{{{\rm{mean}}}}}}^{2}},$$

and ∣Ω∣ is the area of the domain. The algorithm then places the blood vessels in the domain, respecting the experimentally measured minimum distance between vessels, ${\sigma }_{\min }$^74^.

The inhibitor drug enters the domain through the vessel sites and diffuses into the domain, but is also removed at vessel sites along with “wastage” clearance. The following PDE describes the behaviour of the drug concentration, *d*(**x**, *t*), in Ω.

1 $$\frac{\partial d}{\partial t} = \overbrace{D_d \nabla^2 d}^{{{{\rm{diffusion}}}}} - \overbrace{\mu v({{{\mathbf{x}}}}, t) d}^{{{{\rm{clearance}}}}}.$$

*D*_*d*_ is the diffusion coefficient; *μ* is the rate of drug clearance; *v*(**x**, *t*) is the sum of Dirac delta functions centred at vessel sites:

$$v({{{\bf{x}}}},t)=\left\{\begin{array}{ll}1\quad &{{{\rm{if}}}}\;{{{\bf{x}}}}\in {{{\mathcal{V}}}},\\ 0\quad &{{{\rm{otherwise.}}}}\end{array}\right.$$

We mimic drug delivery by imposing maximal drug concentration at vessel sites (effectively acting as Dirichlet boundary conditions). Namely, if the drug is being delivered at time *t*, then we impose *d*(**x**, *t*) = 1 for ${{{\bf{x}}}}\in {{{\mathcal{V}}}}$. Note that *d* ∈ [0, 1] for all **x** and *t*.

We introduce a local density measure *ρ*(**x**) calculated on position **x** in Ω as the sum of the reciprocal exponential Euclidean distance between the position and each blood vessel site (denoted by ${\hat{d}}_{{{{\bf{y}}}}}({{{\bf{x}}}})$):

$$\rho ({{{\bf{x}}}})=\frac{1}{\alpha ({{{\bf{x}}}})}\sum\limits_{{{{\bf{y}}}}\in {{{\mathcal{V}}}}}{e}^{-\hat{\alpha }| | {\hat{d}}_{{{{\bf{y}}}}}({{{\bf{x}}}})| | }.$$

Here $\hat{\alpha }$ is a constant determining the range of each vessel contribution. The normalisation term *α*(**x**) is the sum of the reciprocal exponential Euclidean distance between the position and each location in Ω. We have,

$$\alpha ({{{\bf{x}}}})=\sum\limits_{{{{\bf{y}}}}\in \Omega }{e}^{-\hat{\alpha }| | {\hat{d}}_{{{{\bf{y}}}}}({{{\bf{x}}}})| | }.$$

### Stroma cells

Stroma cells are classified as either reactive or passive. Reactive stroma cells can engage in crosstalk with cancer cells. We define passive stroma as all other cells that do not directly interact with cancer cells (Fig. 1). Stroma cells are randomly initiated as either cell type according to a fixed proportion *π* ∈ [0, 1], where *π* = 0 corresponds to all the stroma being passive and *π* = 1 corresponds to all the stroma being reactive. Reactive stroma cells exist in one of two states: deactivated or activated (Fig. 1 and , respectively). Reactive stroma can change state under specific environmental conditions. Activation occurs with probability *p*_*A*_ in response to recruitment cues from cancer cells. The transition depends on two conditions: the local concentration of the inhibitor drug being above a threshold *h*_*r*_ ∈ [0, 1]; and the reactive stroma cell being proximal (i.e. in the Moore neighbourhood) to a cancer cell to receive the recruitment cues. Here we assume a reactive stroma cell will remain in an activated state as long as the local drug concentration is above *h*_*r*_. We explore alternative hypotheses of reactive stroma deactivation in Supplementary Information S.1.

To recapitulate homeostatic tissue growth, we allow natural turnover of stroma through birth and death processes. Stroma cell death occurs with probability *p*_*T*_. We assume that activation of reactive stroma following therapy-induced wounding/stress signal of cancer cells occurs on a quicker timescale than stroma turnover, hence, *p*_*T*_ is chosen to be one order of magnitude smaller than *p*_*A*_ (Table 1). Unlike cancer cells, stroma cell division is subject to contact inhibition^13^. To model this effect we introduce the parameter ${n}_{CI}\in {\mathbb{N}},$ where cell division of a stroma cell is aborted if there are more than *n*_*CI*_ neighbours in its neighbourhood. Daughter cells are assumed to be of the same cell type (passive or reactive) and state (activated or deactivated) as the mother cell. As a result of turnover and contact inhibition the stroma maintains a dynamical equilibrium. Furthermore, in the absence of cancer, this model can recapitulate tissue repair and homeostasis (Supplementary Information S.8).

### Cancer cells and proliferation signal

To model the increased proliferative and invasive potential of tumours with respect to stroma^11,13^, we do not impose contact inhibition constraints on cancer cells (i.e. a cancer cell will be able to divide as long as there is one empty neighbouring space). Additionally, each newborn cancer cell is assigned an intermitotic time *I* drawn from a uniform distribution calibrated on experimental measures (Table 1). The proliferation signal, *p*(**x**, *t*), determines locally the behaviour and viability of cancer cells (Fig. 1 and , respectively). High local concentrations of the proliferation signal enable growth, hence successful progression through the cell cycle and division. Low values of the proliferation signal can induce quiescence or cell death. We introduce two thresholds *h*_*d*_ and *h*_*p*_, where *h*_*d*_, *h*_*p*_ ∈ [0, 1] and *h*_*d*_ < *h*_*p*_. If *p*(**x**, *t*) < *h*_*d*_, then the local accumulation of signal is not sufficient to ensure viability and any cancer cell at position **x** at time *t* will die. If *p*(**x**, *t*) ≥ *h*_*p*_, then sufficient local pro-growth signalling keeps any cancer cell at position **x** at time *t* in a proliferating state. With intermediate values of the proliferation signal, i.e. *h*_*d*_ ≤ *p*(**x**, *t*) < *h*_*p*_, any cancer cell at position **x** at time *t* will enter a quiescent state, i.e. progression through the cell cycle will be paused. It is well known that there is a lot of heterogeneity amongst cancer cell populations. In this study, as we are focussing specifically on the emergence of EMDR, we do not allow variability in terms of intrinsic sensitivity of cancer cells to the inhibitor drug, and their mutational profile does not evolve.

Cancer cells provide autocrine promotion of the proliferation signal at a rate *β* > 0 (Fig. 1). Paracrine signalling from activated stroma increases *p* at a constant rate *γ* > 0. The inhibitor drug reduces *p* at constant rate *δ* > 0.

The proliferation signal mimics the local accumulation of chemokines, such as growth factors, that are consumed locally or anchored to the ECM^75^, so we do not include any form of movement in space. The proliferation signal is determined by the following PDE:

2 $$\frac{\partial p}{\partial t} = \left(\overbrace{\beta }^{{{{\rm{autocrine}}}}\, {{{\rm{production}}}}} + \, \overbrace{\gamma \sum\limits_{{{{\mathbf{y}}}} \in {{{\mathcal{M}}}}_{{{\mathbf{x}}}}} a({{{\mathbf{y}}}}, t)}^{{{{\rm{paracrine}}}}\, {{{\rm{production}}}}}\right) H(1-p) s({{{\mathbf{x}}}}, t)\, - \overbrace{\delta d p}^{{{{\rm{drug}}}} \, {{{\rm{inhibition}}}}},$$

where ${{{{\mathcal{M}}}}}_{{{{\bf{x}}}}}$ is the Moore neighbourhood of **x**.

Here *s*(**x**, *t*) and *a*(**x**, *t*) are Dirac delta functions centred at cancer sites, and activated stroma sites, respectively:

$$s({{{\bf{x}}}},t)=\left\{\begin{array}{ll}1\quad &{{{\rm{if}}}}\;{{{\bf{x}}}}\in {{{{\mathcal{C}}}}}_{t},\\ 0\quad &{{{\rm{otherwise,}}}}\end{array}\right.$$

and

$$a({{{\bf{x}}}},t)=\left\{\begin{array}{ll}1\quad &{{{\rm{if}}}}\; {{{\bf{x}}}}\in {{{{\mathcal{A}}}}}_{t},\\ 0\quad &{{{\rm{otherwise,}}}}\end{array}\right.$$

where ${{{{\mathcal{C}}}}}_{t}$ and ${{{{\mathcal{A}}}}}_{t}$ are, respectively, the sets of cancer and activated stroma cell locations in Ω at time *t*. The Heaviside function, *H*, models a local saturation of total production once the concentration of *p* reaches 1.

During cell division at each new cancer cell location we assume an initial local proliferation signal of at least *p*_0_, where *h*_*d*_ < *p*_0 _≤ 1.

### Parameter calibration and validation using experimental data

We used Approximate Bayesian Computation to calibrate key model parameters governing the dynamics of tumour growth and response to drug treatment. Firstly, we used experimental data from NSCLC mouse xenografts in a vehicle control cohort (*n* = 10) to infer the joint distribution of tumour growth parameters *p*_0_ and *β* (Supplementary Fig. S3a). Subsequently, we calibrated the rate of drug-induced proliferation signal degradation *δ* with NSCLC mouse xenografts treated with an ALK inhibitor (*n* = 10). The point estimates for *p*_0_, *β* and *δ* reported in Table 1 were chosen from the posterior distributions shown in Supplementary Fig. S3d. Calibration of the rate of paracrine production *γ* was based on in-vitro measurements of the ERK signalling rebound time following administration of a BRAF inhibitor to a tumour/CAF co-culture. Finally, validation of the calibrated model was carried out using additional in-vivo experimental data of growth dynamics following interruption of drug treatment for the same NSCLC mouse xenografts (*n* = 10) treated with the ALK inhibitor drug. Full details of the model parameter calibration and validation can be found in Supplementary Information S.3.

Notably, parameter calibration using in-vivo xenograft mouse models required an adaptation of our in-silico setup to a stroma-free tissue with boundary drug delivery, to match the experimental setup of a subcutaneous tumour mass with extremely limited access to vasculature and stromal tissue. In this in-vitro experimental setup, dynamics operate on a much faster timescale than that of a clinical scenario (weeks versus months, respectively). Remarkably, our fully calibrated model utilised in its original form (i.e. cancer growing within an homeostatic stromal tissue, including a realistic vessel distribution), is able to capture the complex interactions within human tissue, recapitulating dynamics that operate within timescales comparable to those observed in clinical patient data^76^.

### Initial homeostatic tissue, tumour initiation and growth

To create an initial tissue that can be used to investigate tumour growth and the emergence of EMDR upon drug treatment, we first construct a homeostatic bed of stroma cells. Having determined a vessel distribution ${{{\mathcal{V}}}}$ in Ω, all lattice locations not occupied by a vessel are occupied by stroma and the model is run, in the absence of cancer and inhibitor drug, to reach equilibrium. The resulting homeostatic tissue is in a dynamic equilibrium that emerges due to suitably calibrated stromal cell turnover and contact inhibition. The robustness of such a dynamic equilibrium can be tested in response to excessive density and bulk tissue removal (Fig. S3). Our model captures both contact inhibition and wound healing-like behaviour, allowing us to investigate the complexity of cancer-stroma interactions under therapy in a homeostatic tissue.

We initiate cancer by introducing a single cancer cell in the homeostatic tissue, adjacent to a central vessel to mimic a metastatic event. The cancer cell is allowed to proliferate, provided the proliferation signal condition is met, and a tumour is formed in the centre of Ω. In the following we use the configuration of the system when the number of cancer cells reaches 10^4^ (a realistic detection level), as the initial condition for experimentation and analysis of EMDR (Fig. 2b). With this setup, we investigate the complex dynamics that emerge as a result of the multiscale interactions between cancer cells and the TME, shaped by signalling cues and delivery of an inhibitor drug.

### Cell line

EML4-ALK+ H3122 NSCLC cell line were obtained from the Lung Cancer Center of Excellence Cell Line depository at the H. Lee Moffitt Cancer Center. A STR repeats based test was used to authenticate cell lines. For each experiment, a separate frozen vial from an early passage was expanded to generate sufficient numbers of cells for injections and tested for mycoplasma contamination.

### Xenograft tumour model

Xenograft studies were performed by subcutaneous bilateral implantation of 5E6 tumour cells/injection suspended 100 *μ*l of 1: 1 RPMI / BME type 3 (R&D Systems *#*36 − 320 − 1002P) into 4 to 6 week-old NOD-scid IL2Rgnull (NSG) mice of both sexes. The animals were produced at the institute with breeders purchased from Jackson Laboratory. Treatment was initialized 3 weeks post implantation, at which point the tumour diameter reached 3−4 mm. Pharmacological grade Alectinib was dissolved in water and administered via daily oral gavage (7 dats week^−1^) at 25 mgkg^−1^ in 100*μ*l volume. Tumour growth was monitored by weekly electronic caliper measurements. Tumour volumes were calculated assuming spherical shape. All the xenograft studies were performed as per the approved procedures of IACUC protocol *#IS*00005557 of the H. Lee Moffitt Cancer Center. Animals were maintained under AAALAC-accredited specific pathogen-free housing vivarium and veterinary supervision following standard guidelines for temperature and humidity, with a 12-hour light /12-hour dark cycle. Tumour diameter measurements were maintained under the maximal tumour diameter measurement (20 mm) permitted by the IACUC of the H. Lee Mofitt Cancer Center. We have complied with all relevant ethical regulations for animal use.

### Statistics and reproducibility

In-silico results can be reproduced using the hybrid discrete-continuum model code and initial conditions provided^77^ and implemented in Java. The code implements the model with the parameter values given in Table 1. The output data can be used to reproduce cell count (Figs. 2–4), spatial distributions (Figs. 2, 4, 5, 7, 8) and neighbourhood (Figs. 4, 5) graphics. The initial conditions for the domains of the varying vessel densities in Fig. 6 are also provided^77^. Parameter calibration can be reproduced using the ABC model code and data provided^77^ and implemented in Python v3.9.13.

### Reporting summary

Further information on research design is available in the Nature Portfolio Reporting Summary linked to this article.

## Supplementary information

Supplementary Information

Reporting Summary

## Data Availability

The experimental data that support the findings of this study are available at this link^77^. Model output data are available on request.

## Code availability

The code and the simulation results that support the findings of this study are available at this link^77^. The hybrid discrete-continuum model is written in Java and requires the HAL library^65^. The Approximate Bayesian Computation model is written in Python v3.9. 13.
