# Supplementary material for: The role of environmentally mediated drug resistance in facilitating the spatial distribution of residual disease
Source: Commun Biol. 2025 Aug 9;8:1189. doi: 10.1038/s42003-025-08585-9 (PMC12335614; doi:10.1038/s42003-025-08585-9)
Supplement: Supplementary file 1 — Supplementary Information [file 42003_2025_8585_MOESM1_ESM.pdf]

The role of environmentally mediated drug resistance in facilitating the spatial distribution of residual disease. Supplementary Information.

Amy Milne[1], Andriy Marusyk[2], Philip K. Maini[3], Alexander R.A. Anderson[4], and Noemi Picco[1]

[1]Department of Mathematics, Swansea University, Swansea, UK.
[2]Department of Tumor Microenvironment and Metastasis, H. Lee Moffitt Cancer Center, Tampa, FL, USA.
[3]Wolfson Centre for Mathematical Biology, Mathematical Institute, University of Oxford, Oxford, UK.
[4]Department of Integrated Mathematical Oncology, H. Lee Moffitt Cancer Center, Tampa, FL, USA.

July 16, 2025

S.1 Mechanisms of activation and deactivation dynamics for CAFs

We consider alternative hypotheses to the activation and deactivation mechanisms assumed in the model.

Stromal activation hypotheses:

A1) Contact-mediated activation: cross-talk is limited to the tumour-stroma interface, as in the main model.

A2) Non-contact mediated activation: cross-talk acts on a larger range i.e. not limited to interactions in the Moore Neighbourhood.

Stromal deactivation hypotheses:

D1) Active stroma cells deactivate upon loss of condition for activation, i.e. drug concentration falling below h_r, as in the main model.

D2) Active stroma undergoes stochastic death with probability p_T, i.e. active stroma is subject to turnover. Here the reactive stroma acts in a wound healing, immune response, fashion intervening to rescue the cancer under drug treatment, and dying after fulfilling this role.

We investigate combinations of the above hypotheses, for a total of four models. In Figure S1 we compare the current model M1 (A1, D1) with the three additional models emerging from other combinations of activations and deactivation hypotheses: M2 (A1,D2), M3 (A2,D2), and M4 (A2,D1).

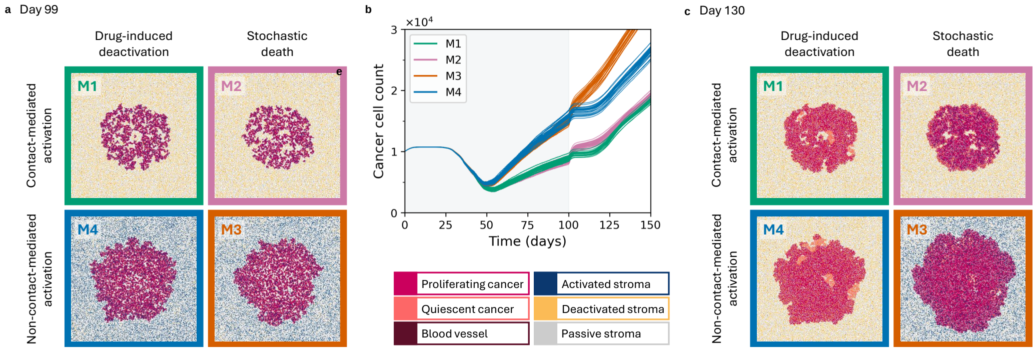

Figure S 1: Comparison of different types of activation and deactivation mechanisms for CAFs. Models M1 (A1,D1), M2 (A1,D2), M3 (A2,D2), and M4 (A2,D1) are considered over 150 days with treatment regime $\tau_T = 100$ days and $\tau_H = 50$ days. **a:** Spatial distributions for a single simulation of each model at day 99, just prior to the end of treatment. **b:** Tumour burden for $t \in [0, 150]$ days. Individual realisations are shown, with 30 stochastic simulations conducted for each model. **c:** Spatial distributions for a single simulation of each model at day 130, after the treatment has stopped.

We first compare the effects of different activation mechanisms on tumour dynamics under treatment. Over 100 days of treatment, non-contact-mediated activation (A2) results in greater tumour burden (Fig. S1**b**). This is due to stromal activation across the whole domain, and cancer cells proliferating into activated regions having a greater chance of survival from the paracrine assistance. Conversely, contact-mediated activation (A1) limits paracrine assistance, hence survival, to the tumour periphery, resulting in reduced and less densely packed growth (Fig. S1**a**). While different deactivation mechanisms do not have a significant effect on the treatment period, a clear separation of dynamics occurs when treatment is stopped (Fig. S1**b**). Stochastic death (D2) operates independently of the dynamics of drug leaving the tissue. Deactivation takes longer, and residual active stroma continues to provide paracrine assistance well into the drug holiday period (Fig. S1**c**), enhancing tumour regrowth. Non-contact-mediated activation (A2) further augments regrowth, compared to

contact-mediated activation (A1), as cancer cells proliferate into 'primed' regions of activated stroma outside of the tumour boundary, and receive additional paracrine assistance.

S.2 Intermittent Treatment

We investigate intermittent treatment regimes $\tau_H = 10$ days, $\tau_H = 20$ days and $\tau_H = 30$ days with $\tau_T = \{0, 10, 20, 30, 40, 50, 60, 70, 80, 90, 100\}$ days. Cumulative days of drug delivery decrease as τ_H increases for each choice of τ_T and relative tumour burden increases as τ_H increases for each choice of τ_T (Fig. S2**a**). For $\tau_T < 50$ days there is significant residual disease present at the end of the first drug delivery period, almost always signifying treatment failure. Tumour regrowth in holiday periods does not happen for intermittent treatment regimes with $\tau_H = 10$ days (Fig. S2**b**), whereas for intermittent treatment regimes where $\tau_H > 10$ days there is tumour regrowth in the holiday period that is consistent with experimental results[1]. As τ_H increases the regrowth during drug holiday periods also increases, resulting in overall higher amounts of residual disease. Figure S2**c** shows tumour regrowth during the drug holiday period for $\tau_H = 20$ days and Figure S2**d** shows greater tumour regrowth for $\tau_H = 30$ days. We choose intermittent regime $\tau_T = 50$ days and $\tau_H = 20$ days for spatial analysis.

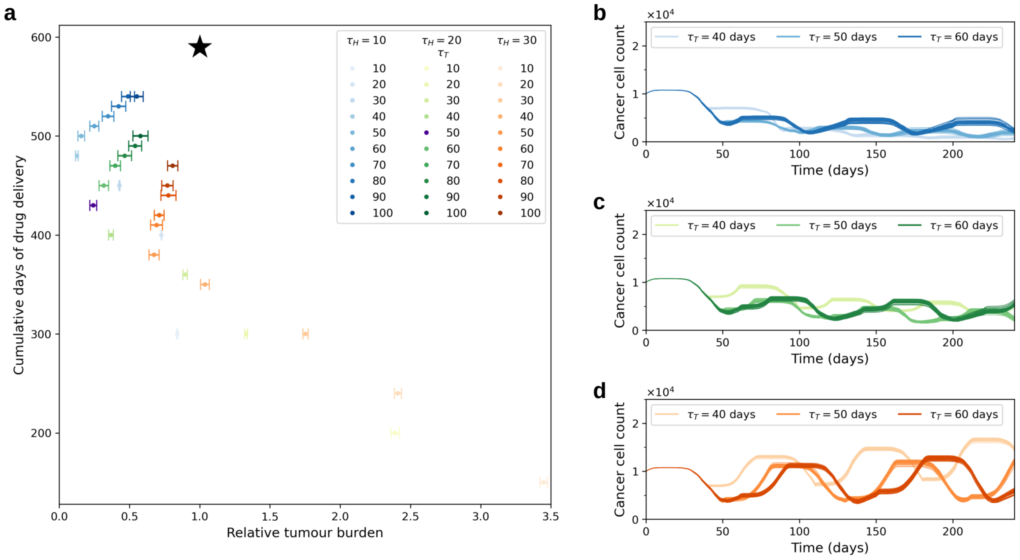

Figure S2: Investigation of treatment regimes. a Cumulative days of drug delivery (measured as the sum of drug delivery days over 590 days of therapy) against relative tumour burden (measured as the sum of total cancer cell count over the $t \in [0, 590]$ day window normalised to the continuous treatment case) for $\tau_T = \{10, 20, 30, 40, 50, 60, 70, 80, 90, 100\}$ days for $\tau_H = 10$ days (*blue*), $\tau_H = 10$ days (*green*), and $\tau_H = 30$ days (*orange*). Results show the average of 30 simulations of each schedule and includes 95% confidence intervals. The star indicates measures for continuous treatment. As τ_H increases the relative tumour burdens for each τ_T also increases and while the cumulative days of treatment decreases. Relative tumour burden initially decreases as cumulative days of drug delivery increases, but from; $\tau_T = 40$ days for the $\tau_H = 10$ days regime; and $\tau_T = 50$ days for the $\tau_H = \{20, 30\}$ days regimes; the relative tumour burden increases. **b - d** show tumour burden over 30 simulations for treatment regimes $\tau_T = [40, 50, 60]$ days from day 0 to day 240. **b:** $\tau_H = 10$ days. **c:** $\tau_H = 20$ days. **d:** $\tau_H = 30$ days. These intermittent treatment schedules show, overall, a long-term improved outcome compared to continuous treatment (Figure 2).

S.3 Parameter Calibration

We use in-vivo xenograft model data provided by the Marusyk Lab to calibrate the growth of cancer cells in the absence of treatment and the initial rate of decay when undergoing molecularly targeted therapy. The data provided are for a xenograft model of ALK+ non-small cell lung cancer (NSCLC), the H3122 cells subcutaneously injected into flanks of five NSG mice ($n = 10$), for the vehicle control and treatment

(ALK inhibitor - alectinib) groups. Tumour growth was monitored based on caliper-based measurements.

Human NSCLC cells subcutaneously injected into mouse xenograft models have very different growth dynamics to that of the same cells in human tissue. In order to replicate the experimental setup we remove stroma cells from our model and increase the division radius as we expect no infiltration of non-cancerous cells and reduced spatial constraints in the xenograft model during the experiment time frame. As an initial condition for the calibration we place a single cancer cell in the centre of the domain which is allowed to proliferate in ideal conditions ($p_0 = h_p$ and $\beta = 1$) until a tumour consisting of 10^4 cells is formed. We then use this configuration to first calibrate the growth dynamics of the tumour against the control data (untreated xenografts). Specifically, we use Approximate Bayesian Computation (ABC) to refine a uniform joint distribution for the initial local proliferation signal value assigned to cancer cells at division, p_0, and the rate of autocrine production, β, identifying a posterior distribution that matches the experimental data. Once these parameters are calibrated we then perform ABC to calibrate the rate of drug inhibition, δ, using the ALK treatment data. Details of the implementation of ABC have been described previously[2].

S.3.1 Initial local proliferation signal p_0 and the rate of autocrine production β

We assume that the initial local proliferation signal p_0 does not exceed the threshold for proliferation h_p as a cell with a $p_0 \geq h_p$ does not require the additional autocrine signalling in order to divide. Hence we impose the upper bound $p_0 < h_p$.

Increasing β has a saturating effect on growth dynamics: once β is sufficiently high, p quickly reaches h_p and tumour growth depends on spatial constraints only. We

choose a maximum value of β that is greater than this saturation point determined by experimentation.

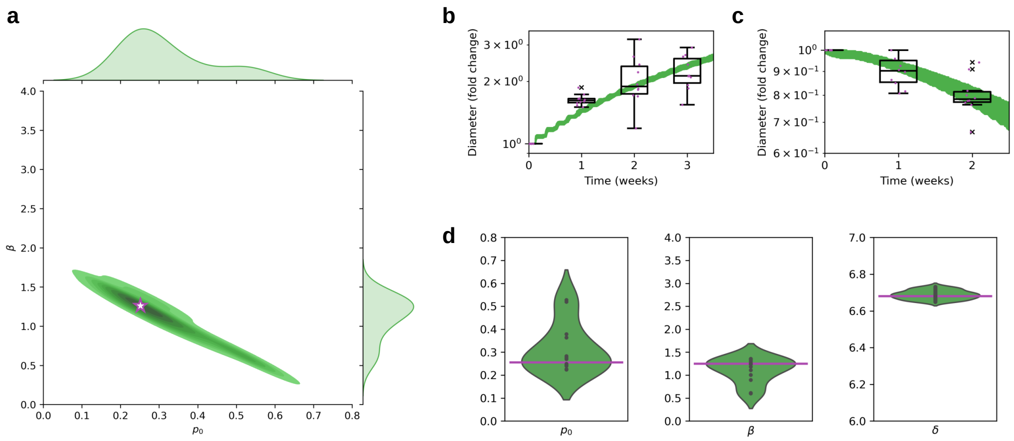

Figure S3: Experimental calibration of p_0, β, and δ. a: Joint and marginal posterior distributions for p_0 and β obtained by Approximate Bayesian Computation (ABC) with $N = 10^4$ and $\epsilon = 0.018$, showing inverse correlation between p_0 and β accepted values. The star marks the chosen point estimates: $p_0 = 0.256$, $\beta = 1.25$. **b:** Diameter fold change for in-vivo experimental data ($n = 10$ vehicle control replicates, ***black*** boxplots and ***purple*** data points.) and in-silico tumours (***green*** time-series for parameter regimes corresponding to the (p_0, β) posterior distribution). **c:** Diameter fold change for in-vivo experimental data ($n = 10$ drug-treated replicates, ***black*** boxplots and ***purple*** data points) and in-silico tumours (***green*** time-series for parameter regimes corresponding to the δ posterior distribution). For the boxplots in **b** and **c** centre line is the median; box limits are the upper and lower quartiles; whiskers are $\pm 1.5 \times$ IQR; and outliers are represented by a cross. **d:** Marginal posterior distributions for p_0 and β (corresponding to the posterior in **a**), and posterior distribution for δ obtained by ABC with $N = 10^4$ and $\epsilon = 0.0005$. Horizontal lines mark the chosen point estimates.

We use uniform priors: $(p_0, \beta) \in (U[0, h_p] \times U[0, 4])$, discard pairs that result in an intermitotic time greater than 2 days, and perform ABC with $N = 10^4$ and $\epsilon = 0.018$. The ABC identifies a range of inversely correlated p_0 and β values (Fig. S3**a**). This is consistent with what we expect: high initial local pro-growth signalling requires lower autocrine production to sustain survival and proliferation, whereas a higher amount of autocrine signalling is required for the cancer cell to survive and proliferate if the

initial local pro-growth signalling is low. In the posterior distribution we identify a peak corresponding to a p_0 value just above h_d and a β value over 1. We take these as point estimates ($p_0 = 0.256$, $\beta = 1.25$ day^{-1}) ensuring a good fit of the in-silico tumour growth dynamics to the vehicle control experimental cohort (Fig. S3**b**).

S.3.2 Rate of drug inhibition δ

To recapitulate the subcutaneous in-vivo experimental set up for the drug treated cohort we impose maximal drug at the periphery of the tumour and allow it to diffuse into the tumour. Initial scanning of the tumour decay dynamics for a range of δ values enables us to identify a suitable uniform prior: $\delta \in U[6, 7]$. ABC is then performed with $N = 10^4$ and $\epsilon = 0.0005$, to calibrate the drug-induced tumour reduction against the in-vivo experimental data (Fig. S3**c**). Within the resulting posterior distribution, we chose point estimate for parameter δ which corresponds to the peak ($\delta = 6.68$ day^{-1}).

Violin plots of the posterior distributions for the tumour growth parameters p_0 and β, and the tumour decay parameter δ are shown in Figure S3**d**, indicating the chosen point estimates for each parameter.

S.3.3 Rate of paracrine production γ

To calibrate the rescue effect of CAFs, we estimate the rate of paracrine production γ using the in-vitro experimental data[3]. These results show that melanoma-associated fibroblasts (the experimental counterpart to our reactive stroma cells) confer tolerance to the inhibitor drug, restoring ERK signalling within 12 hours. We define this time to recovery as t_R, which in our modelling framework corresponds to the average time it takes cancer cells in stromal co-cultures targeted with the drug to replenish their proliferation signal. Matching this average time to

the experimental measure, we can calibrate parameter γ as follows.

Consider a cancer cell hit by treatment when experiencing the proliferation signal at the threshold h_d at time t_0. Eq. (2) can be simplified to consider the worst case scenario of the inhibitor drug concentration at maximum concentration ($d = 1$) and minimum paracrine help (one out of eight neighbouring activated reactive stroma cells), to obtain:

$$\frac{\partial p}{\partial t} = \beta + \gamma - \delta p,$$

which has solution:

$$p(t) = \frac{\beta + \gamma}{\delta} + \left(h_d - \frac{\beta + \gamma}{\delta}\right) e^{-\delta(t-t_0)} := \theta(h_d, \gamma, t - t_0) \text{ for } t > t_0.$$

For the single activated stroma cell to be effective in rescuing a cancer cell from its death fate, it must push the local proliferation signal above h_p. Hence, we obtain a lower bound on the paracrine production rate, $\underline{\gamma}$, imposing:

$$\theta(h_d, \underline{\gamma}, t_R) \geq h_p,$$

Note that this condition cannot be solved explicitly for γ, so the lower bound, $\underline{\gamma}$, on the paracrine production rate is determined numerically. Additionally, note that $\underline{\gamma}$ depends on the choice of β and δ. With our choice of $\beta = 1.25$ and $\delta = 6.68$, the numerical lower bound $\underline{\gamma} = 4.2412$, so we choose $\gamma = 4.242$ day^{-1} cell^{-1}.

S.3.4 Validation of the calibrated model

We validate the chosen parameter regime by comparing the output of our calibrated model to the regrowth dynamics of additional in-vivo experimental data. Tumour diameters of the NSCLC mouse xenografts ($n = 10$) treated with ALK inhibitor (experimental setup described in S.3) are measured for four weeks after stopping treatment. We match this experiment in-silico by simulating a drug delivery period followed by a period where drug delivery is ceased and the drug is allowed to diffuse out of the boundary and the vasculature. The time-series profiles of the tumour diameter fold change for 30 simulations match well the in-vivo experimental data for the regrowth period, thus validating the parameter calibration (Fig. S4).

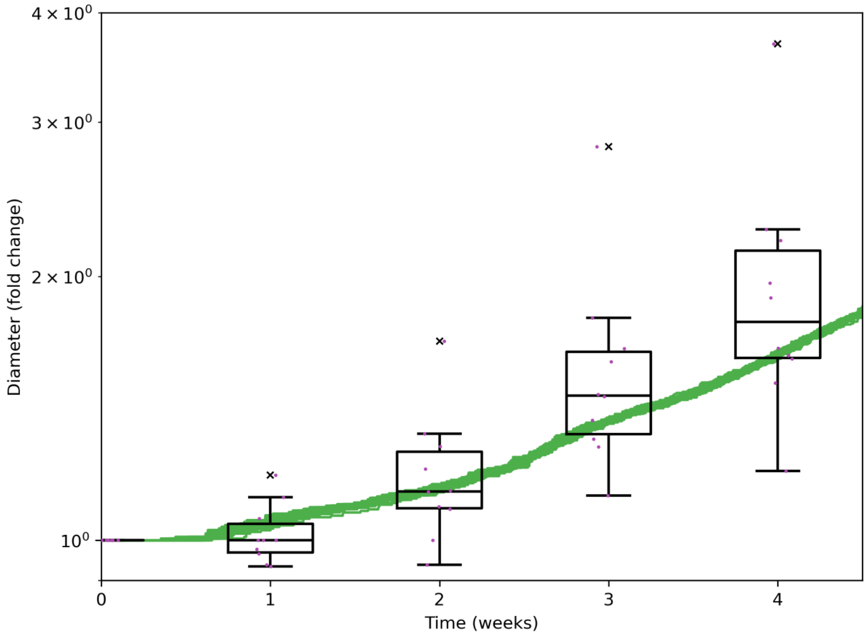

Figure S4: Validation of calibrated parameters to in-vivo data. Tumour diameter fold change for in-silico tumours (***green*** time-series for 30 simulations of the model with $p_0 = 0.256$, $\beta = 1.25$ and $\delta = 6.68$) compared to the in-vivo experimental data ($n = 10$ drug-treated replicates post treatment, ***black*** boxplots and ***purple*** data points). For the boxplots centre line is the median; box limits are the upper and lower quartiles; whiskers are $\pm 1.5 \times$ IQR; and outliers are represented by a cross.

S.4 Cancer Neighbourhoods

We analyse the distribution of cancer cell neighbours of cancer cells in the configuration of cells in Ω taken as the initial condition for in-silico experiments (Figure 2**b**, the details of how this is constructed is described in the Initial homeostatic tissue, tumour initiation and growth section). There is a slight negative skew with an average of 5 cancer neighbours in their Moore neighbourhood (Fig. S5).

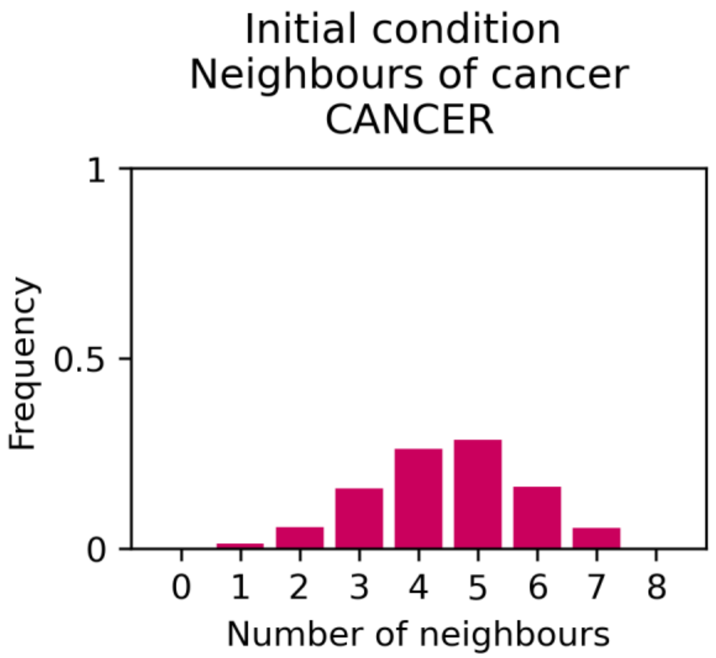

Figure S 5: Distribution of cancer neighbours of cancer cells in the initial condition. The distribution of cancer neighbours of cancer cells in the initial condition has a slight negative skew with an average of five cancer neighbours.

The distribution of reactive stroma neighbours of cancer cells for each of the identified niches (survival, eradication and persistance) is shown in Figure S6. Here the survival niche has more reactive stroma neighbours of surviving cancer cells than both the eradication and persistence niches.

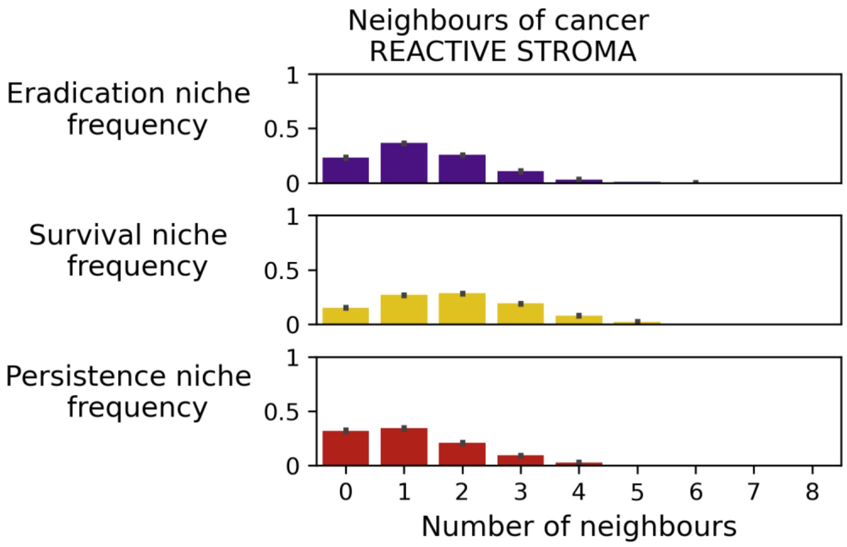

Figure S6: Distribution of average reactive stroma neighbours of cancer cells. Distribution of average reactive stroma neighbours of cancer cells in the survival niche (yellow), the eradication niche (purple) and the persistence niche (red) from day 0 to day 590 for the same 30 simulations as Figure 4 with standard errors shown. The survival, eradication and persistence niches are the same as those in Figure 5.

S.5 Animations

The following animations can be viewed at **this link**:

- No treatment.
- Continuous treatment.
- Intermittent treatment regime with $\tau_T = 10$ days, $\tau_H = 20$ days.
- Intermittent treatment regime with $\tau_T = 20$ days, $\tau_H = 20$ days.
- Intermittent treatment regime with $\tau_T = 30$ days, $\tau_H = 20$ days.
- Intermittent treatment regime with $\tau_T = 40$ days, $\tau_H = 20$ days.
- Intermittent treatment regime with $\tau_T = 50$ days, $\tau_H = 20$ days.

- Intermittent treatment regime with $\tau_T = 60$ days, $\tau_H = 20$ days.
- Intermittent treatment regime with $\tau_T = 70$ days, $\tau_H = 20$ days.
- Intermittent treatment regime with $\tau_T = 80$ days, $\tau_H = 20$ days.
- Intermittent treatment regime with $\tau_T = 90$ days, $\tau_H = 20$ days.
- Intermittent treatment regime with regime $\tau_T = 100$ days, $\tau_H = 20$ days.
- proliferation signal and drug concentrations for survival and eradication niches for intermittent treatment regime with $\tau_T = 50$ days, $\tau_H = 20$ days.

S.6 Vessel Density Test

Nine distinct domains are created with 50, 150, 200, 250, 300, 350, 400 and 450 vessel sites respectively. The corresponding mean field ρ values are 0.54×10^{-3}, 1.63×10^{-3}, 2.18×10^{-3}, 2.73×10^{-3}, 3.28×10^{-3}, 3.82×10^{-3}, 4.38×10^{-3} and 4.94×10^{-3} respectively. There is a scaling applied to the σ_{min} for each domain to ensure adequate dispersal of vessels and no clustering. Each domain then has a homogeneous bed of stroma created on which a cancer cell can be placed and a tumour grown as the initial condition. A treatment regime of 50 days treatment and 20 days holiday is simulated for 200 days. Figure S7**a** shows the tumour burden for varying mean field ρ. We observe a non-linear relationship between tumour burden and mean field ρ. For low mean field ρ tumour burden is high (poor perfusion failure) and as mean field ρ increases we have a diminishing tumour burden until mean field $\rho = 3.82 \times 10^{-3}$ where the tumour burden then increases again as mean field ρ continues to increase (environmentally mediated drug resistance). The emergence of different patterns of treatment failure dependant on mean field ρ is shown in Figure S7**b**. Low mean field ρ exhibits features of treatment failure due to poor perfusion of the inhibitor drug in the domain. Here the tumour mass is maintained

throughout and we observe the increased fitness of cancer over stroma. For high mean field ρ we observe waves of cell death with small resistant populations. Here there is less competition between cancer and stroma cells as stroma cells are able to proliferate into regions vacated by the cancer death wave. These dynamics suggest that constraints on local drug delivery could affect treatment efficacy.

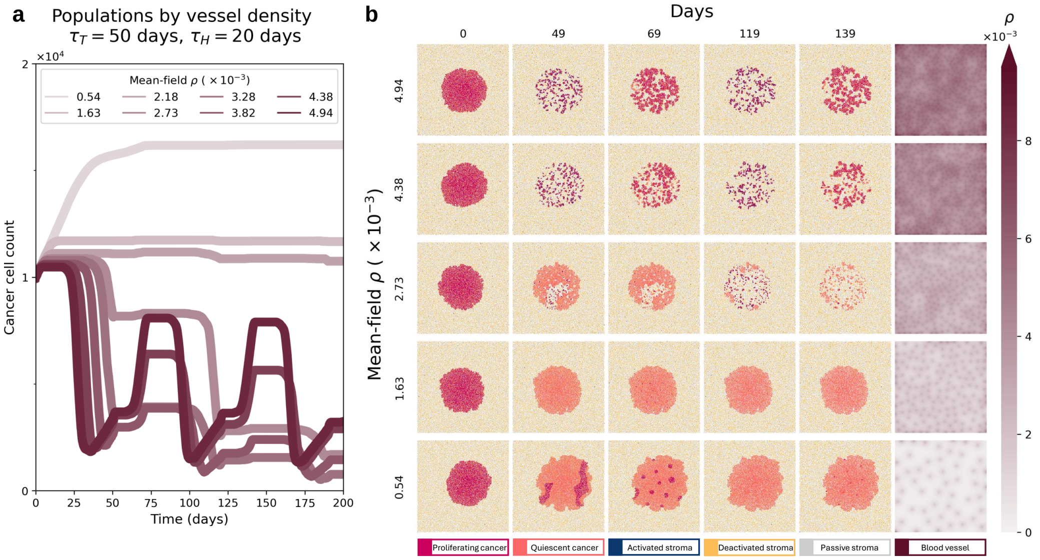

Figure S7: Vessel Density Measure Investigation. a: Tumour burden for each mean field ρ equal to 0.54×10^{-3}, 1.63×10^{-3}, 2.18×10^{-3}, 2.73×10^{-3}, 3.28×10^{-3}, 3.82×10^{-3}, 4.38×10^{-3} and 4.94×10^{-3} for 200 days. **b:** The first five columns show visualisation of cells for single representative simulations for mean field ρ equal to 0.54×10^{-3}, 1.63×10^{-3}, 2.73×10^{-3}, 4.38×10^{-3} and 4.94×10^{-3} on day 0, 49, 69, 119 and 139. Vessel sites are the same as those in Figure 6. The last column shows visualisation of the vessel density measure ρ for each vessel field considered. Here $\hat{\alpha} = 0.1$. As ρ increases the spatial attributes of the treatment failure change. At lower ρ treatment failure is driven by poor perfusion where the inhibitor drug is not able to reach sufficient concentration levels to reduce the proliferation signal below h_d. For higher ρ, where the inhibitor drug is able to perfuse and build up in the domain, environmentally mediated drug resistance is evident where cancer survivors are clustered around activated stroma.

S.7 Numerical Implementation

From Eq. (1) we write the forward difference scheme in time and second order central difference scheme is space as:

$$\begin{aligned} {d_{i,j}}^{q+1} = {d_{i,j}}^{q}\left[1 - \Delta t\left(\mu {v_{i,j}}^{q} + \frac{4D_d}{(\Delta x)^2}\right)\right] \\ + \Delta t\left[\frac{D_d}{(\Delta x)^2}\left({d_{i-1,j}}^{q} + {d_{i,j-1}}^{q} + {d_{i+1,j}}^{q} + {d_{i,j+1}}^{q}\right)\right]. \end{aligned}$$

To ensure the stability of this explicit numerical scheme we need to impose a constraint on Δt. We consider the vessel sites in the worst case scenario of no diffusion from neighbouring positions. Since we are to avoid the case where $d < 0$, we have:

$$1 - \Delta t\left(\mu + \frac{4D_d}{(\Delta x)^2}\right) \geq 0.$$

This results in an upper bound on Δt:

$$\Delta t \leq \frac{1}{\mu + \frac{4D_d}{(\Delta x)^2}}.$$

The upper bound on Δt only applies to the drug concentration dynamics and it is not necessary for other compartments of the model to operate on such a fine grain. Hence, we update cell and proliferation signal dynamics every ten Δt to improve model efficiency.

S.8 Regeneration of tissue

We test the homeostatic bed of stroma cells in response to excessive density and bulk tissue removal. We create two insets in the homeostatic tissue where the stroma cells are replaced: one with densely packed stroma cells and the other empty of stroma cells (Fig. S8**a**). The system is evolved through time using the model and we observe the densely packed inset becoming less dense and stroma cells infiltrating the empty inset mimicking the phenomena of contact inhibition-driven tissue homeostasis and the process of wound healing (Fig. S8**b**). After sufficient time both insets exhibit the same homeostatic state within a fully homogeneous tissue (Fig. S8**c,d**).

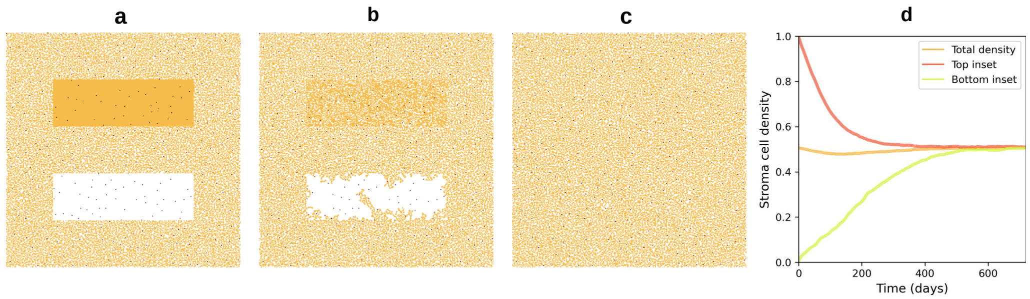

Figure S8: Tissue regeneration and homeostasis occur as a result of stroma turnover and contact inhibition. a-c: Progression towards homeostasis of stroma tissue at day 0 (**a**), day 96 (**b**) and day 720 (**c**) of a single representative simulation of the model in the absence of cancer and inhibitor drug. There are two insets at day 0, one packed with stroma where there are no empty lattice positions and the other with all stroma removed. By day 96 the packed inset is visibly less dense as the phenomenon of contact inhibition prohibits further stroma proliferation. In the empty inset, we observe stroma cells infiltrating, mimicking the closure of a wound. At day 720 there is no trace of the original insets, rather a homeostatic bed of stroma. **d:** Stroma cell density for each inset and the whole domain from day 0 to day 720.

References

[1] Vander Velde, R. et al. Resistance to targeted therapies as a multifactorial, gradual adaptation to inhibitor specific selective pressures. *Nat Commun* **11**,

2393 (2020).

[2] Picco, N., Sahai, E., Maini, P.K. & Anderson, A.R.A. Integrating models to quantify environment-mediated drug resistance. *Cancer Res* **77(19)**, 5409-5418 (2017).

[3] Hirata, E. et al. Intravital imaging reveals how BRAF inhibition generates drug-tolerant microenvironments with high integrin β1/FAK signaling. *Cancer Cell* **27(4)**, 574-588 (2015).
